# Adjuvant icotinib for resected EGFR-mutated stage II–IIIA non-small-cell lung cancer (ICTAN, GASTO1002): a randomized comparison study

**DOI:** 10.1038/s41392-025-02358-w

**Published:** 2025-08-28

**Authors:** Ning Li, Wei Ou, Chao Cheng, Jian You, Lin Yang, Feng-Xia Chen, Yi Liang, Zhixiong Yang, Bao-Xiao Wang, Zeng-Hao Chang, Yao-Bin Lin, Weixiong Yang, Feng Xu, Guanggui Ding, Xian-Shan Chen, Ronggui Hu, Shujun Li, Hao Jiang, Xin-Xin Hu, Hao Long, Si-Yu Wang

**Affiliations:** 1 State Key Laboratory of Oncology in South China, Guangdong Provincial Clinical Research Center for Cancer, Sun Yat-sen University Cancer Center, Guangzhou, China; 2 The First Affiliated Hospital of Sun Yat-sen University, Guangzhou, China; 3 Tianjin Medical University Cancer Institute & Hospital, National Clinical Research Center for Cancer, Tianjin’s Clinical Research Center for Cancer, Tianjin, China; 4 Shenzhen People’s Hospital (The Second Clinical Medical College, Jinan University, the First Affiliated Hospital of South University of Science and Technology of China), Shenzhen Institute of Respiratory Diseases, Shenzhen, China; 5 Hainan General Hospital, Haikou, China; 6 Zhongshan City People’s Hospital, Zhongshan, China; 7 Affiliated Hospital of Guangdong Medical University, Zhanjiang, China; 8 Sun Yat-sen Memorial Hospital, Sun Yat-sen University, Guangzhou, China; 9 Present Address: Guangdong Provincial Key Laboratory of Cancer Pathogenesis and Precision Diagnosis and Treatment, Shenshan Medical Center, Sun Yat-sen Memorial Hospital, Sun Yat-sen University, Shanwei, China

**Keywords:** Cancer therapy, Lung cancer

## Abstract

The efficacy, safety and ideal treatment duration of an adjuvant epidermal growth factor receptor tyrosine kinase inhibitor (EGFR-TKI) for patients with resected EGFR-mutated non-small-cell lung cancer (NSCLC) were not known until 2014, when this study was initiated. In this phase 3 ICTAN trial (GASTO1002, NCT01996098), patients with completely resected, EGFR-mutated, stage II-IIIA NSCLC after adjuvant chemotherapy were assigned in a 1:1:1 ratio to receive icotinib (125 mg, three times daily) for 12 months, to receive icotinib for 6 months, or to undergo observation. The primary endpoint was disease-free survival (DFS). This trial was terminated early. A total of 251 patients were randomized. Adjuvant icotinib for 12 months significantly improved DFS (hazard ratio [HR]: 0.40, 95% confidence interval [CI], 0.27–0.61; *P* < 0.001) and overall survival (OS; HR: 0.55, 95% CI, 0.32–0.96; *P* = 0.032) compared with observation. Adjuvant icotinib of 6 months also significantly improved DFS (HR: 0.41, 95% CI, 0.27–0.62; *P* < 0.001) and OS (HR: 0.56, 95% CI, 0.32–0.98; *P* = 0.038) compared with observation. Adjuvant icotinib for 12 months did not improve DFS (HR: 0.97; *P* = 0.89) or OS (HR: 1.00; *P* = 0.99) compared with 6 months of this drug. Rates of adverse events of grade 3 or higher were 8.3%, 6.0% and 2.4% for the 12-month icotinib, 6-month icotinib, and observation groups, respectively. Adjuvant icotinib for 12 months or 6 months following adjuvant chemotherapy improved DFS and OS compared with observation in patients with resected EGFR-mutated stage II-IIIA NSCLC with a manageable safety profile, supporting it as a potential treatment option.

## Introduction

Lung cancer is the most frequently diagnosed cancer and the leading cause of cancer mortality worldwide.^1^ Approximately 2.5 million newly diagnosed lung cancer cases and 1.8 million lung cancer deaths were reported in 2022 worldwide.^1^ China is encountering a rising burden of lung cancer, with 1.0 million new cases and 0.7 million lung cancer deaths in 2022, accounting for 43% of global newly diagnosed lung cancer cases and 40% of global lung cancer deaths, respectively.^2^ Non-small-cell lung cancer (NSCLC) accounts for about 85% of all lung cancers, and most patients are diagnosed at a later stage.^3^ Approximately 30% of patients with NSCLC present with resectable disease at the time of diagnosis.^4,5^ For patients with resectable NSCLC, one of the standard-of-care treatments is surgery with curative intent, followed by adjuvant platinum-based chemotherapy for patients with stage II-IIIA and selected stage IB disease.^6^ However, chemotherapy is associated with only a 5.4% absolute survival improvement at 5 years, and the risk of disease recurrence or death remains high for patients with resectable NSCLC.^7^ Although great improvements have been made in the diagnosis and treatment of NSCLC, the long-term survival outcomes are still unsatisfactory, with the 5-year survival rate ranging from 68% for stage IIA disease to 50% for stage IIIA disease.^8^

Great progress has been achieved in the treatment of NSCLC over the past two decades with the discovery of multiple crucial oncogenic drivers and the development of corresponding targeted therapies. Mutations of epidermal growth factor receptors (EGFR) occur more frequently in East-Asia patients (about 50% for lung adenocarcinoma) than in Western patients (10–15%).^9,10^ EGFR tyrosine kinase inhibitors (TKIs) have markedly altered the therapeutic landscape of NSCLC, leading to the development of targeted therapies that have improved patient outcomes. EGFR-TKIs have been established for patients with advanced NSCLC harboring EGFR mutations, with 3 generations of TKIs showing improvement in progression-free survival (PFS) compared with chemotherapy.^3^ Based on the success in the advanced stage, EGFR-TKIs were assessed in the adjuvant setting. Two early studies (BR19 and RADIANT) investigating the value of EGFR-TKIs as adjuvant therapies did not select patients based on EGFR mutational status.^11,12^ In both studies, no survival benefit was found after 2 years of a first-generation EGFR-TKI. In the single-arm SELECT study, encouraging disease-free survival (DFS) results (2-year DFS of 88%) were obtained after 2 years of erlotinib for resectable EGFR-mutated NSCLC.^13,14^ Moreover, in our phase II randomized trial evaluating gefitinib following chemotherapy as adjuvant therapy in patients with resected EGFR-mutated stage IIIA-N2 NSCLC, the addition of 6-month gefitinib significantly prolonged DFS compared with chemotherapy alone.^15^ The results of these studies preliminarily demonstrate the potential of EGFR-TKIs with or without chemotherapy in adjuvant therapy for resected NSCLC with EGFR mutations.

Icotinib is an EGFR-TKI of the first generation, which was approved for the treatment of advanced EGFR-mutated NSCLC based on the results from the ICOGEN and the CONVINCE trials.^16,17^ Results from the ICOGEN study indicated that icotinib was non-inferior to gefitinib in previously treated advanced NSCLC in terms of PFS (median, 4.6 months vs. 3.4 months), OS (median, 13.3 months vs. 13.9 months), and objective response rate (27.6% vs. 27.2%). Of note, patients in the icotinib group had fewer drug-related adverse events (AEs) than those in the gefitinib group (61% vs. 70%).^16^ In the CONVINCE trial, first-line icotinib significantly improves PFS compared with chemotherapy (median PFS, 11.2 months vs. 7.9 months) in patients with advanced EGFR-mutated lung adenocarcinoma, with a manageable safety profile.^17^ Extending the use of icotinib to the adjuvant setting was evaluated in the EVIDENCE trial, which demonstrated that 2 years of icotinib significantly prolonged DFS compared with adjuvant chemotherapy (median DFS, 47.0 months vs. 22.1 months) for resected EGFR-mutated stage II-IIIA NSCLC.^18^ However, the EVIDENCE trial was designed to compare icotinib with standard chemotherapy, and chemotherapy was not included in the experimental group. The efficacy, safety, and ideal treatment duration of EGFR-TKIs following adjuvant chemotherapy for patients with completely resected EGFR-mutated NSCLC were not known until 2014, when this study was initiated.

We hypothesized that icotinib following adjuvant chemotherapy would be more effective in reducing the risk of disease recurrence than chemotherapy alone for resected EGFR-mutated NSCLC in the adjuvant setting. This open-label, phase 3, randomized ICTAN trial (ClinicalTrials.gov identifier: NCT01996098) investigated whether icotinib following adjuvant chemotherapy improves long-term survival outcomes compared with observation in stage II-IIIA NSCLC with EGFR mutations. We also evaluated the safety and tolerability of icotinib after adjuvant chemotherapy in these patients.

## Results

### Patient characteristics

**Fig. 1:**
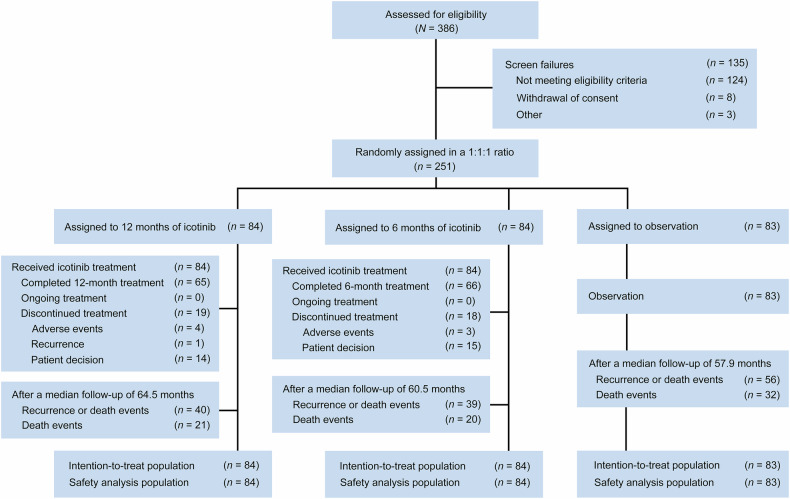
Trial profile. All randomized patients were included in the ITT population for efficacy analyses. Safety analyses were performed for all patients who received icotinib and those in the observation group. ITT, intention-to-treat; N number

From July 2014 through December 2021, 386 patients were screened for enrollment, and a total of 251 patients were randomly assigned, with 84 patients in the 12-month icotinib group, 84 patients in the 6-month icotinib group, and 83 patients in the observation group (Fig. 1). This trial was terminated early owing to slow accrual and the publication of EVIDENCE and ADAURA data. The baseline characteristics were generally well balanced between the groups: more than 60% of the patients were female, more than 85% were never smokers, and 59% had stage IIIA disease (Table 1). Almost all the patients had adenocarcinoma. For surgery, lobectomy was performed in the majority of the patients.

**Table 1:** Baseline characteristics of the intention-to-treat population

	Icotinib for 12 months (*n* = 84)	Icotinib for 6 months (*n* = 84)	Observation (*n* = 83)
Age (years), median (minimum, maximum)	57 (34–75)	56 (33–75)	57 (24–75)
Sex			
Male	30 (35.7)	29 (34.5)	30 (36.1)
Female	54 (64.3)	55 (65.5)	53 (63.9)
ECOG PS			
0	41 (48.8)	43 (51.2)	38 (45.8)
1	40 (47.6)	39 (46.4)	42 (50.6)
Missing	3 (3.6)	2 (2.4)	3 (3.6)
Smoking status			
Never	72 (85.7)	73 (86.9)	72 (86.7)
Former	5 (6.0)	6 (7.1)	5 (6.0)
Current	7 (8.3)	5 (6.0)	6 (7.2)
Histology			
Adenocarcinoma	83 (98.8)	83 (98.8)	83 (100.0)
Other	1 (1.2)	1 (1.2)	0
Stage^a^			
IIA	6 (7.1)	5 (6.0)	5 (6.0)
IIB	28 (33.3)	29 (34.5)	29 (34.9)
IIIA	50 (59.5)	50 (59.5)	49 (59.0)
N stage			
N0	9 (10.7)	8 (9.5)	8 (9.6)
N1	30 (35.7)	29 (34.5)	29 (34.9)
N2	45 (53.6)	47 (56.0)	46 (55.4)
Surgery type			
Lobectomy	82 (97.6)	84 (100.0)	81 (97.6)
Bilobectomy	1 (1.2)	0	1 (1.2)
Pneumonectomy	1 (1.2)	0	1 (1.2)
Side			
Left	39 (46.4)	36 (42.9)	40 (48.2)
Right	45 (53.6)	48 (57.1)	43 (51.8)
EGFR mutation			
Exon 19 deletion	46 (54.8)	47 (56.0)	44 (53.0)
Exon 21 L858R	38 (45.2)	37 (44.0)	39 (47.0)

Data are presented as *n* (%) unless stated otherwise

*ECOG PS* Eastern Cooperative Oncology Group performance status, *EGFR* epidermal growth factor receptor, *AJCC* American Joint Committee on Cancer

^a^Staging was determined according to the 7th edition of the AJCC TNM staging system for lung cancer

For adjuvant chemotherapy, all four treatment cycles were completed in 68 (81.0%) patients in the 12-month icotinib group, 66 (78.6%) patients in the 6-month icotinib group, and 67 (80.7%) patients in the observation group. Three cycles of adjuvant chemotherapy were completed in 6, 7, and 7 patients in each group, respectively. In total, 70% of the patients received pemetrexed and carboplatin as adjuvant chemotherapy. A summary of the adjuvant chemotherapy cycles and regimens is provided in Supplementary Table 1.

For treatment compliance, 65 patients (77.4%) in the 12-month icotinib group completed the planned 12-month treatment, and 66 patients (78.6%) in the 6-month icotinib group completed the planned 6-month treatment. In the 12-month icotinib group, 19 patients discontinued icotinib treatment because of patient decisions (*n* = 14), AEs (*n* = 4), or recurrence (*n* = 1). In the 6-month icotinib group, 18 patients discontinued icotinib treatment because of patient decisions (*n* = 15) and AEs (*n* = 3). The median duration of treatment was 12.1 months (interquartile range [IQR], 11.7–12.5) in the 12-month icotinib group and 6.5 months (IQR, 6.0–7.0) in the 6-month icotinib group (Fig. 1). None of the patients in the icotinib groups were still receiving the study medication at the time of analysis.

### Efficacy

**Fig. 2:**
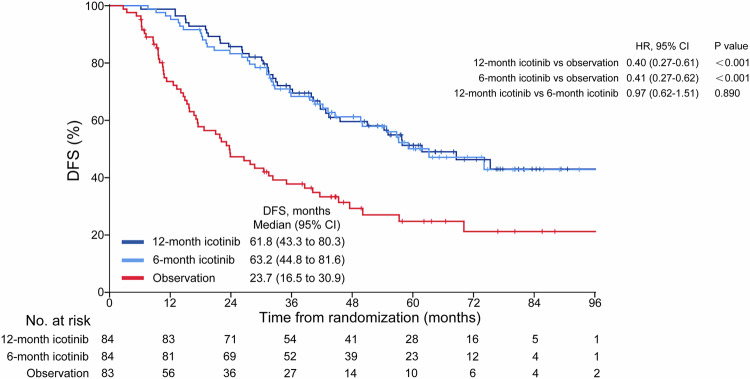
Kaplan-Meier estimates of DFS by the investigator in the ITT population. DFS disease-free survival, HR hazard ratio, CI confidence interval, ITT intention-to-treat

**Fig. 3:**
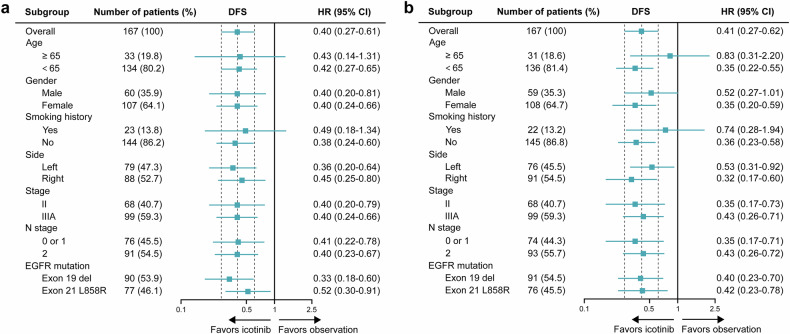
Forest plots of DFS by subgroup, according to investigator assessment. **a** Subgroup analyses of DFS for 12-month icotinib versus observation. **b** Subgroup analyses of DFS for 6-month icotinib versus observation. Subgroups were either pre-planned (sex, stage, and EGFR mutation type) or post hoc (age, smoking history, N stage, and side). DFS disease-free survival, HR hazard ratio, CI confidence interval, EGFR epidermal growth factor receptor

The data cutoff date was January 15, 2024. The median duration of follow-up in the intention-to-treat (ITT) population was 61.4 months (IQR, 44.9–79.8). The median follow-up for DFS was 64.5 months in the 12-month group, 60.5 months in the 6-month group, and 57.9 months in the observation group. By the time of the analysis, there had been 135 disease recurrence or death events. There were 40 (47.6%) disease relapse or death events in the 12-month group, 39 (46.4%) in the 6-month group, and 56 (67.5%) in the observation group. Adjuvant icotinib of 12 months significantly improved DFS (hazard ratio [HR]: 0.40, 95% confidence interval [CI], 0.27–0.61; log-rank *P* < 0.001; stratified log-rank *P* < 0.001) compared with observation (Fig. 2). Icotinib of 6 months also significantly improved DFS (HR: 0.41, 95% CI, 0.27–0.62; log-rank *P* < 0.001; stratified log-rank *P* < 0.001) compared with observation. Adjuvant icotinib of 12 months did not improve DFS (HR: 0.97; log-rank *P* = 0.89; stratified log-rank *P* = 0.87) compared with 6 months of this drug (Fig. 2). The median DFS was 61.8 (95% CI, 43.3 to 80.3) months for the 12-month icotinib group, 63.2 (95% CI, 44.8 to 81.6) months for the 6-month icotinib group compared with 23.7 (95% CI, 16.5 to 30.9) months for the observation group. The 5-year DFS for the 12-month icotinib, 6-month icotinib, and observation groups were 51.3%, 50.1% and 24.8%, respectively (Fig. 2). The estimated statistical power based on the actual number of enrolled patients was 0.76 for 12 months of icotinib versus observation, and 0.72 for 6 months of icotinib versus observation. In the per-protocol population, adjuvant icotinib of 12 months (HR: 0.41, 95% CI, 0.26–0.64; log-rank *P* < 0.001) or 6 months (HR: 0.41, 95% CI, 0.26–0.64; log-rank *P* < 0.001) significantly improved DFS compared with observation (Supplementary Fig. 1). Adjuvant icotinib of 12 months did not improve DFS (HR: 1.00; log-rank *P* = 0.997) compared with 6 months of icotinib (Supplementary Fig. 1). The DFS benefit of 12-month icotinib over observation was generally consistent across most subgroups, including in the patients with either stage II or stage IIIA disease, those with either N0/N1 or N2 disease, and those who had exon 19 deletion or exon 21 L858R mutation (Fig. 3a). In addition, the DFS benefit of 6-month icotinib over observation was also observed in most subgroups (Fig. 3b). However, no differences in efficacy pertaining to icotinib duration were found in any of the subgroups (Supplementary Table 2). Among the patients with stage II disease, the 5-year DFS for the 12-month icotinib, 6-month icotinib and observation groups were 56.9% (95% CI, 38.9 to 74.9), 55.9% (95% CI, 33.9 to 77.9) and 29.3% (95% CI, 10.9 to 47.7); respectively (Supplementary Fig. 2a). Among the patients with stage IIIA disease, the 5-year DFS for the 12-month icotinib, 6-month icotinib and observation groups were 47.5% (95% CI, 32.4 to 62.6), 45.1% (95% CI, 30.6 to 59.6) and 21.9% (95% CI, 9.0 to 34.8); respectively (Supplementary Fig. 2b).

In the overall population, the lung was the most common first site of relapse, which occurred in 11 (13.1%), 12 (14.3%), and 20 (24.1%) patients in the 12-month icotinib, 6-month icotinib, and observation groups, respectively (Supplementary Tables 3 and 4). The brain was the second most common first site of relapse, which occurred in 10 (11.9%), 12 (14.3%), and 15 (18.1%) in the 12-month icotinib, 6-month icotinib, and observation groups, respectively (Supplementary Tables 3 and 4). The 12-month icotinib group had a significantly improved brain-metastasis-free survival (BMFS) compared with the observation group, with a 5-year BMFS of 68.5% versus 54.5% (HR: 0.53, 95% CI, 0.32–0.88; log-rank *P* = 0.012; Supplementary Fig. 3). Icotinib of 6 months was also associated with an improved BMFS compared to observation, with the 5-year BMFS of 68.4% versus 54.5% (HR: 0.54, 95% CI, 0.33–0.90; log-rank *P* = 0.016; Supplementary Fig. 3). After disease relapse, 29, 29, and 44 patients in the 12-month, 6-month, and observation groups received a first subsequent therapy, respectively (Supplementary Table 5). Most patients received an EGFR-TKI-based treatment as their first subsequent therapy, including 23 (57.5%), 26 (66.7%), and 43 (76.8%) patients who experienced recurrence in the 12-month, 6-month, and observation groups, respectively. Overall, 17 (42.5%) patients in the 12-month icotinib group, 12 (30.8%) patients in the 6-month icotinib group, and 9 (16.1%) patients in the observation group received a third-generation EGFR-TKI as the first subsequent therapy (Supplementary Table 5). However, there was no significant difference in the rate of patients receiving third-generation EGFR-TKIs for all subsequent therapies, with 25 (62.5%), 25 (64.1%), and 32 (57.1%) patients receiving a third-generation EGFR-TKI in the 12-month, 6-month, and observation groups, respectively (Supplementary Table 6).

**Fig. 4:**
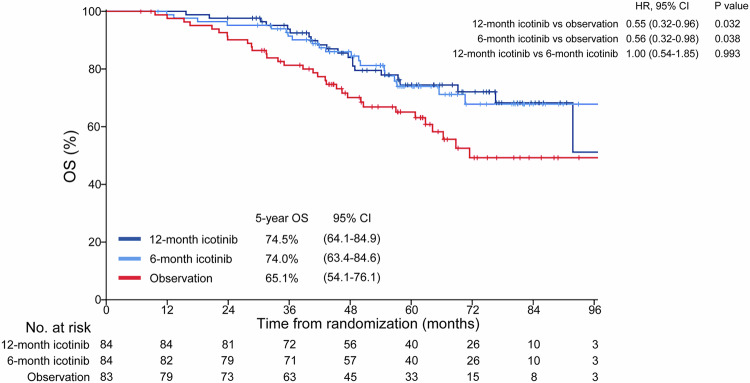
Kaplan-Meier estimates of OS in the ITT population. OS, overall survival; HR, hazard ratio; CI, confidence interval; ITT, intention-to-treat

By the time of the analysis, 73 death events had occurred, including 21 in the 12-month icotinib group, 20 in the 6-month icotinib group, and 32 in the observation group. Overall survival (OS) was significantly more prolonged among the patients in the 12-month icotinib group than among those in the observation group (HR: 0.55, 95% CI, 0.32–0.96; log-rank *P* = 0.032; Fig. 4). OS was also significantly longer among those who had been assigned to the 6-month icotinib group than among those in the observation group (HR: 0.56, 95% CI, 0.32–0.98; log-rank *P* = 0.038; Fig. 4). However, adjuvant icotinib of 12 months did not improve OS compared with 6 months of this drug (HR: 1.00, 95% CI, 0.54–1.85; log-rank *P* = 0.99; Fig. 4). The 5-year OS for the 12-month, 6-month and observation groups were 74.5%, 74.0% and 65.1%, respectively. In the per-protocol population, significantly longer OS was observed in the 12-month group (HR: 0.53, 95% CI, 0.29–0.97; log-rank *P* = 0.036) than the observation group (Supplementary Fig. 4). Adjuvant icotinib of 6 months demonstrated a marginal OS benefit (HR: 0.56, 95% CI, 0.31–1.03; log-rank *P* = 0.058) compared with observation (Supplementary Fig. 4). Adjuvant icotinib of 12 months did not prolong OS compared with 6 months of this drug (HR: 0.96; log-rank *P* = 0.90; Supplementary Fig. 4). There were no significant differences between 12-month or 6-month icotinib and observation in most of the subgroups (Supplementary Fig. 5a, b), possibly because the study was not powered to examine OS benefit within the subgroups.

### Safety

A summary of the AEs is provided in Supplementary Table 7. AEs of any grade occurred in 65 (77.4%) patients in the 12-month group, 62 (73.8%) in the 6-month group, and 35 (42.2%) in the observation group. The most common AEs at any grade were rash (36.9%), diarrhea (21.4%), and raised aminotransferases (14.3%) in the 12-month group, and rash (38.1%), diarrhea (19.0%), and raised aminotransferases (13.1%) in the 6-month group, and leukopenia (8.4%), insomnia (8.4%), and raised aminotransferases (4.8%) in the observation group (Table 2).

**Table 2:** Most common all-cause AEs reported in ≥5% of patients

	12-month icotinib (*n* = 84)		6-month icotinib (*n* = 84)		Observation (*n* = 83)	
All-cause AEs	Any grade	Grade ≥ 3	Any grade	Grade ≥ 3	Any grade	Grade ≥ 3
Total^a^	65 (77.4)	7 (8.3)	62 (73.8)	5 (6.0)	35 (42.2)	2 (2.4)
Rash	31 (36.9)	3 (3.6)	32 (38.1)	2 (2.4)	1 (1.2)	0
Diarrhea	18 (21.4)	1 (1.2)	16 (19.0)	1 (1.2)	3 (3.6)	0
Raised aminotransferases	12 (14.3)	1 (1.2)	11 (13.1)	0	4 (4.8)	0
Leukopenia	4 (4.8)	1 (1.2)	5 (6.0)	0	7 (8.4)	1 (1.2)
Insomnia	6 (7.1)	1 (1.2)	4 (4.8)	1 (1.2)	7 (8.4)	0
Fatigue	5 (6.0)	0	4 (4.8)	1 (1.2)	3 (3.6)	1 (1.2)
Decreased appetite	4 (4.8)	0	5 (6.0)	0	2 (2.4)	0
Oral ulcers	5 (6.0)	0	3 (3.6)	0	1 (1.2)	0

Data are presented as n (%). All AEs were reported in at least 5% of the patients in either trial group, according to the National Cancer Institute Common Terminology Criteria for Adverse Events, version 4.0

^a^Total number of patients who had at least one AE; some patients had more than one AE

Abbreviations: AEs, adverse events

Grade 3 or worse AEs were reported for 7 (8.3%), 5 (6.0%), and 2 (2.4%) patients in the 12-month, 6-month, and observation groups, respectively. The grade 3 or worse AEs experienced in the 12-month group were rash in 3 patients (3.6%), diarrhea in 1 (1.2%), raised aminotransferases in 1 (1.2%), leukopenia in 1 (1.2%), and insomnia in 1 (1.2%). The grade 3 or worse AEs that occurred in the 6-month group were rash in 2 patients (2.4%), diarrhea in 1 (1.2%), insomnia in 1 (1.2%), and fatigue in 1 (1.2%). The grade 3 or worse AEs that occurred in the observation group were leukopenia in 1 patient (1.2%) and fatigue in 1 (1.2%; Table 2). AEs leading to permanent treatment discontinuation were recorded in 4 (4.8%) and 3 (3.6%) patients in the 12-month and 6-month groups, respectively. No interstitial lung disease (ILD) was reported in either group. No treatment-related deaths or dose reductions occurred in either group (Supplementary Table 7). No new safety events have been observed since the previous reports for icotinib.

## Discussion

This phase 3 open-label randomized ICTAN trial demonstrated a significant DFS benefit with 12-month or 6-month icotinib compared to observation in patients with completely resected EGFR-mutated stage II–IIIA NSCLC after adjuvant chemotherapy. The DFS benefit of 12- or 6-month icotinib over observation was generally consistent across most subgroups. The ICTAN trial also showed an improvement in OS and BMFS with icotinib versus observation in these patients. However, adjuvant icotinib of 12 months did not improve DFS, OS, or BMFS compared with 6 months of this drug.

The DFS noted among the patients treated with icotinib (median DFS, 61.8 months for the 12-month group and 63.2 months for the 6-month group) in the ICTAN trial was notably longer than that reported in the EVIDENCE trial (median DFS, 47.0 months).^18^ The results of this study also revealed an OS benefit of adjuvant icotinib in resected EGFR-mutated stage II–IIIA NSCLC. DFS benefit did not consistently translate into a significant difference in OS for first-generation EGFR-TKIs.^18–21^ The OS benefit in ICTAN may be largely attributed to the study design of 100% adjuvant chemotherapy. Most randomized trials (including CTONG1104, EVIDENCE, and IMPACT) evaluating adjuvant EGFR-TKIs were designed to compare an EGFR-TKI with standard chemotherapy; thus, they did not include chemotherapy in the experimental group.^18,21–23^ Although the impact of adjuvant chemotherapy was not addressed by the study design in ADAURA,^24^ the 5-year OS was higher among the stage II-IIIA patients with adjuvant chemotherapy than among those without chemotherapy in the osimertinib arm (87% vs. 80%) as well as in the placebo arm (75% vs. 66%),^25^ suggesting a potential survival benefit with adjuvant chemotherapy in this population. Thus, adjuvant chemotherapy, which is associated with a 16% reduction in the risk of recurrence or death in NSCLC (HR for disease recurrence or death, 0.84),^7^ remains necessary for EGFR-mutated stage II-IIIA NSCLC when an EGFR-TKI was administered.^26^ Another possible reason for the OS benefit might be the sequential synergistic effect of chemotherapy and EGFR-TKIs. In vitro data suggest that chemotherapy combined with an EGFR-TKI may act in concert in EGFR-mutated NSCLC.^27^ The FLAURA2 trial indicated that the combination of osimertinib and chemotherapy improved PFS over osimertinib alone as first-line treatment for EGFR-mutated advanced NSCLC.^28^ Sequential use of chemotherapy and an EGFR-TKI as adjuvant treatment to eliminate minimal residual disease may be a rational option for resected EGFR-mutated NSCLC. In addition, the low crossover rate of EGFR-TKIs in the observation group may also influence the OS in our study. After a DFS event, only 76.8% (43/56) of patients received EGFR-TKIs as subsequent treatment in the observation group in our study.

Researchers noted that the improvement in OS seen with osimertinib was not consistently observed with earlier generation EGFR-TKIs,^18–21,25^, and this difference may be owing to the ability to inhibit EGFR T790M mutation and/or the superior intracranial activity of osimertinib. Although icotinib does not inhibit the T790M mutation, it is interesting to note that BMFS was improved with icotinib in the current trial. In the BRAIN trial, icotinib prolonged intracranial PFS compared with whole-brain irradiation plus chemotherapy (median intracranial PFS, 10.0 months vs. 4.8 months).^29^ Practically, early introduction of icotinib in the adjuvant setting could provide the opportunity for patients to receive the most effective first-line third-generation EGFR-TKI after recurrence. Although more patients in the 12- or 6-month icotinib group than in the observation group received a third-generation EGFR-TKI as their first subsequent therapy in the current study (42.5%, 30.8%, and 16.1%, respectively), the use of third-generation EGFR-TKIs for all subsequent therapies was generally balanced among the three groups (62.5%, 64.1% and 57.1%, respectively). In other words, patients who experienced progression with first-generation EGFR-TKIs received third-generation EGFR-TKIs ultimately, suggesting the feasibility of early introduction of first-generation EGFR-TKIs. In safety analyses, compared with the AEs reported in ADAURA, the AEs in the current study were lower; grade 3 or worse AEs were reported to be 23% in the osimertinib arm in ADAURA,^25^ and 6% to 8% in the icotinib arms in our study. Notably, ILD was recorded in 3% of patients in ADAURA, whereas no ILD occurred in our study. These results indicate that icotinib can be a treatment option to reduce the risk of brain metastases and improve survival in resected EGFR-mutated NSCLC with favorable safety.

Various durations of EGFR-TKIs treatment have been investigated in clinical studies. Adjuvant osimertinib for 3 years is recommended for patients with resected EGFR-mutated NSCLC.^24,25^ The phase II ICOMPARE study demonstrated that 2 years of icotinib improved DFS and OS in patients with stage II-IIIA lung adenocarcinoma without chemotherapy, compared with 1 year of this treatment.^30^ In EVIDENCE, the treatment duration of icotinib was set to be 2 years.^18^ However, whether 3, 2, or 1 year, or even half of a year, is the optimal duration for adjuvant EGFR-TKIs remains to be determined due to the lack of formal testing in head-to-head, randomized studies. Thus, a key question is whether the survival benefit can be prolonged after discontinuation of the trial regimen. From the results of our trial, adjuvant 6 months of icotinib following chemotherapy seemed to be enough for completely resected EGFR-mutated NSCLC to avoid overtreatment. For first-generation EGFR-TKIs, drug resistance develops after 6–12 months of treatment, and more than 50% of the resistance is associated with the EGFR T790M mutation.^31,32^ Moreover, after 1–2 years of osimertinib (targeting T790M mutation) treatment, new challenging resistance mechanisms eventually emerge.^31^ In the CORIN trial, adjuvant icotinib for 1 year led to a significant increase of 3-year DFS compared with observation (96.1% vs. 84.0%) for stage IB NSCLC with EGFR mutations.^33^ Compared with the stage IB subgroup in ADAURA, it seems that the CORIN trial achieved a similar clinical benefit with a first-generation EGFR-TKI and reduced treatment duration. With the results of the current study combined with those above, it should be highlighted that de-escalation of adjuvant TKI therapy might be feasible for completely resected EGFR-mutated NSCLC after adjuvant chemotherapy. Thus, efficacy, cost, toxicity, and treatment compliance should be taken into account in the selection of an EGFR-TKI and treatment duration in the adjuvant therapy of completely resected EGFR-mutated NSCLC after adjuvant chemotherapy. Circulating tumor DNA has shown its potential value in identifying patients who are at high risk of recurrence after resection,^34^ and may facilitate individualized treatment in the adjuvant setting.

Multiple phase III trials assessing the role of an EGFR-TKI for resected EGFR-mutated NSCLC are currently ongoing, including ICWIP (NCT02125240), APEX (NCT04762459), FORWARD (NCT04853342), ADAURA2 (NCT05120349), and BD-BF-III01 (NCT06041776). The ongoing ICWIP study compares 3-year icotinib versus placebo after adjuvant chemotherapy for EGFR-mutated resected NSCLC. We look forward to future clinical trials of personalized postoperative adjuvant EGFR-TKI for resected NSCLC.

The main limitation of our study was its early termination (due to slow accrual and the publication of EVIDENCE and ADAURA data), which led to a relatively small sample size and made the study underpowered for subgroup analysis in OS. Another limitation is that this study was delayed more than expected. During this trial, several treatment practices changed for localized and advanced EGFR-mutated NSCLC. Other limitations include the non-blinded study design and the fact that 100% of the patients were of Asian ethnicity. Despite these limitations, our data provide crucial evidence on adjuvant first-generation icotinib for patients with resected EGFR-mutated NSCLC after adjuvant chemotherapy.

In conclusion, the findings of the ICTAN trial demonstrated that icotinib for 12 or 6 months prolonged DFS, OS, and BMFS compared with observation for patients with completely resected stage II-IIIA EGFR-mutated NSCLC after adjuvant chemotherapy. Furthermore, icotinib was well tolerated, and no new safety issues were observed. These results support first-generation icotinib as a potential treatment option for these patients after adjuvant chemotherapy. De-escalation of adjuvant TKI therapy following chemotherapy is feasible for completely resected EGFR-mutated NSCLC.

## Materials and methods

### Study design and participants

The ICTAN (GASTO1002) trial is a phase 3, randomized, open-label, multicenter trial conducted at 7 centers in China. Eligible patients were adult males or females (aged 18–75 years) with postsurgical pathological stage II-IIIA NSCLC (as classified according to the 7th edition of the American Joint Committee on Cancer TNM staging system); having completed at least two cycles of adjuvant platinum-based chemotherapy; having a centrally confirmed EGFR mutation (exon 19 deletion and/or exon 21 L858R). Additional eligibility criteria were an Eastern Cooperative Oncology Group performance status (ECOG PS) score of 0 or 1, adequate hematological function, adequate liver and renal function, and a life expectancy of at least 1 year.

Patients with a second primary malignancy in the past 5 years (except for cured basal cell carcinoma of the skin or cured in situ carcinoma of the uterine cervix); any prior systemic antitumor treatment other than adjuvant chemotherapy; previous radiotherapy; any unstable systemic disease (such as unstable heart disease or uncontrolled hypertension); or a history of ILD were ineligible for this study. R0 resection of the lung cancer was mandatory (the definition of R0 is provided in the trial protocol in the **Supplementary information**). Additional eligibility criteria are provided in the trial protocol.

### Ethics approval and consent to participate

This trial was conducted in accordance with the provisions of the Declaration of Helsinki, Good Clinical Practice guidelines, and applicable laws and regulations. The protocol and any amendments were approved by the medical ethical committee of the Guangdong Association of Study of Thoracic Oncology (GASTO) and the independent ethics committee at each trial site. This report was prepared in accordance with the 2025 Consolidated Standards of Reporting Trials (CONSORT) reporting guideline.^35^ The CONSORT 2025 checklist is available in the Supplementary information. All participants provided written informed consent to participate. This trial was registered with ClinicalTrials.gov on November 27, 2013, with the number NCT01996098.

### Randomization and blinding

The randomization scheme was produced by the central staff of the GASTO via a computer-generated sequence with a minimization method. Randomization was stratified according to sex (male vs. female) and stage (II vs. IIIA). Eligible patients were randomized at a ratio of 1:1:1 to receive icotinib for 12 months, icotinib for 6 months, or to undergo observation. All patients, investigators, and staff involved in the study were unblinded to the treatment assignment.

### Procedures

After completing chemotherapy, patients were randomized at a 1:1:1 ratio to receive icotinib (125 mg, three times daily) for 12 months, icotinib for 6 months, or to undergo observation. The treatment continued until study completion, disease recurrence, intolerable toxicity, or death.

Follow-up assessments were scheduled every 3 months for the first 2 years after randomization, every 6 months until 5 years, and every 12 months thereafter until disease relapse or death. The follow-up assessments comprised contrast-enhanced computed tomography (CT) of the chest and abdomen, brain magnetic resonance imaging (MRI), and bone scans. Brain MRI and bone scans were scheduled every 12 months or performed as indicated based on symptoms. Recurrence was evaluated according to the Response Evaluation Criteria in Solid Tumors (RECIST), version 1.1, by the investigator. Post-recurrence therapy, which was chosen by investigators, was permitted.

Safety assessments were performed from randomization to every visit. AEs were graded according to the National Cancer Institute’s Common Terminology Criteria for Adverse Events (NCI-CTCAE), version 4.0. AEs were managed according to the AE management protocol. Quality of life (QoL) was assessed with the Functional Assessment of Cancer Therapy—Lung Cancer (FACT-L) questionnaire and the Lung Cancer Symptom Scale (LCSS).

### Endpoints

The primary endpoint was DFS according to investigator assessment. DFS was defined as the duration from random assignment to disease recurrence or death, whichever occurs first. The secondary end points included OS (defined as the time from random assignment to death from any cause), BMFS (defined as the time from randomization to brain metastasis or death, whichever comes first), safety, and QoL. QoL outcomes will be reported elsewhere.

### Statistical analysis

On the basis of previous studies,^7,11^ we assumed that the median DFS is 30 months for patients with EGFR-mutated stage II-IIIA NSCLC following adjuvant chemotherapy. We aimed to achieve 85% power at a two-sided α of 0.05 and an overall dropout rate of 5%. A total of 318 patients were needed to detect a 40% improvement in DFS with icotinib compared with chemotherapy for each comparison: 6 months of icotinib versus observation and 12 months of icotinib versus observation. This improvement corresponded to an HR of 0.6. A total of 198 DFS events were required for the final analysis.

All analyses for efficacy were based on the ITT population, which included all randomized patients. Safety analyses were performed for all patients who received icotinib and those in the observation group. The per-protocol population included patients who completed planned 12 or 6 months of icotinib. For efficacy comparisons, the Kaplan-Meier method was used to estimate time-to-event endpoints, with differences compared by log-rank tests with two-sided *p* values. A Cox proportional hazards model was used to estimate HRs, 95% CIs, and Wald *P* values. A pre-planned subgroup analysis of DFS was performed for sex (male vs. female), stage (II vs. IIIA), and EGFR mutation type (exon 19 deletion vs. exon 21 L858R). The subgroup analysis of age (≥65 vs. <65), smoking history (ever vs. never), N stage (0 and 1 vs. 2), and side (left vs. right) was post hoc. Full details of statistical analyses are provided in the statistical analysis plan (available in the **Supplementary information**).

## Supplementary information

Supplementary Materials

Trial Protocol

Statistical Analysis Plan

CONSORT 2025 Checklist

## Data Availability

The authenticity of this article has been validated by uploading the key raw data onto the Research Data Deposit public platform (www.researchdata.org.cn), with the approval RDD number as RDDA2025495496. The datasets used and/or analyzed during the current study are available from the Lead Contact, Si-Yu Wang, on a reasonable request.
