# Supplementary material for: Adjuvant icotinib for resected EGFR-mutated stage II–IIIA non-small-cell lung cancer (ICTAN, GASTO1002): a randomized comparison study
Source: Signal Transduct Target Ther. 2025 Aug 28;10:273. doi: 10.1038/s41392-025-02358-w (PMC12391381; doi:10.1038/s41392-025-02358-w)
Supplement: Supplementary file 1 — Supplementary Materials [file 41392_2025_2358_MOESM1_ESM.docx]

Supplementary Materials for

Adjuvant icotinib for resected EGFR-mutated stage II–IIIA non-small cell lung cancer (ICTAN, GASTO1002): a randomized comparison study

Ning Li, Wei Ou, Chao Cheng, Jian You, Lin Yang, Feng-Xia Chen, Yi Liang, Zhixiong Yang, Bao-Xiao Wang, Zeng-Hao Chang, Yao-Bin Lin, Weixiong Yang, Feng Xu, Guanggui Ding, Xian-Shan Chen, Ronggui Hu, Shujun Li, Hao Jiang, Xin-Xin Hu, Hao Long & Si-Yu Wang.

Correspondence: Si-Yu Wang (wangsy@gasto.org.cn) or Hao Long (longhao@sysucc.org.cn).

**This PDF file includes:**

Supplementary Figures 1 to 5

Supplementary Tables 1 to 7


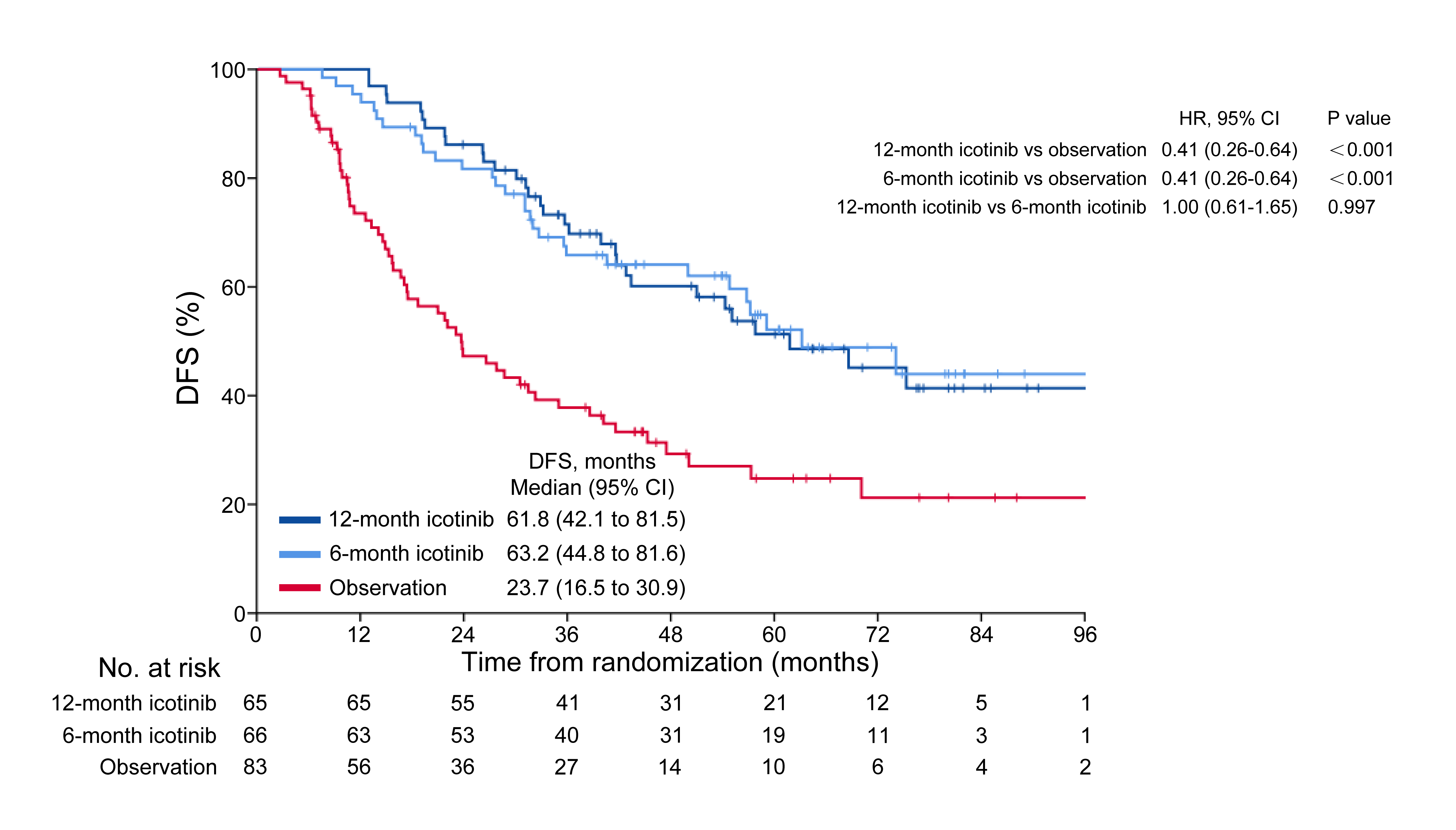


Supplementary Figure 1.

Kaplan-Meier estimates of DFS by the investigator in the per-protocol population. DFS, disease-free survival; HR, hazard ratio; CI, confidence interval.


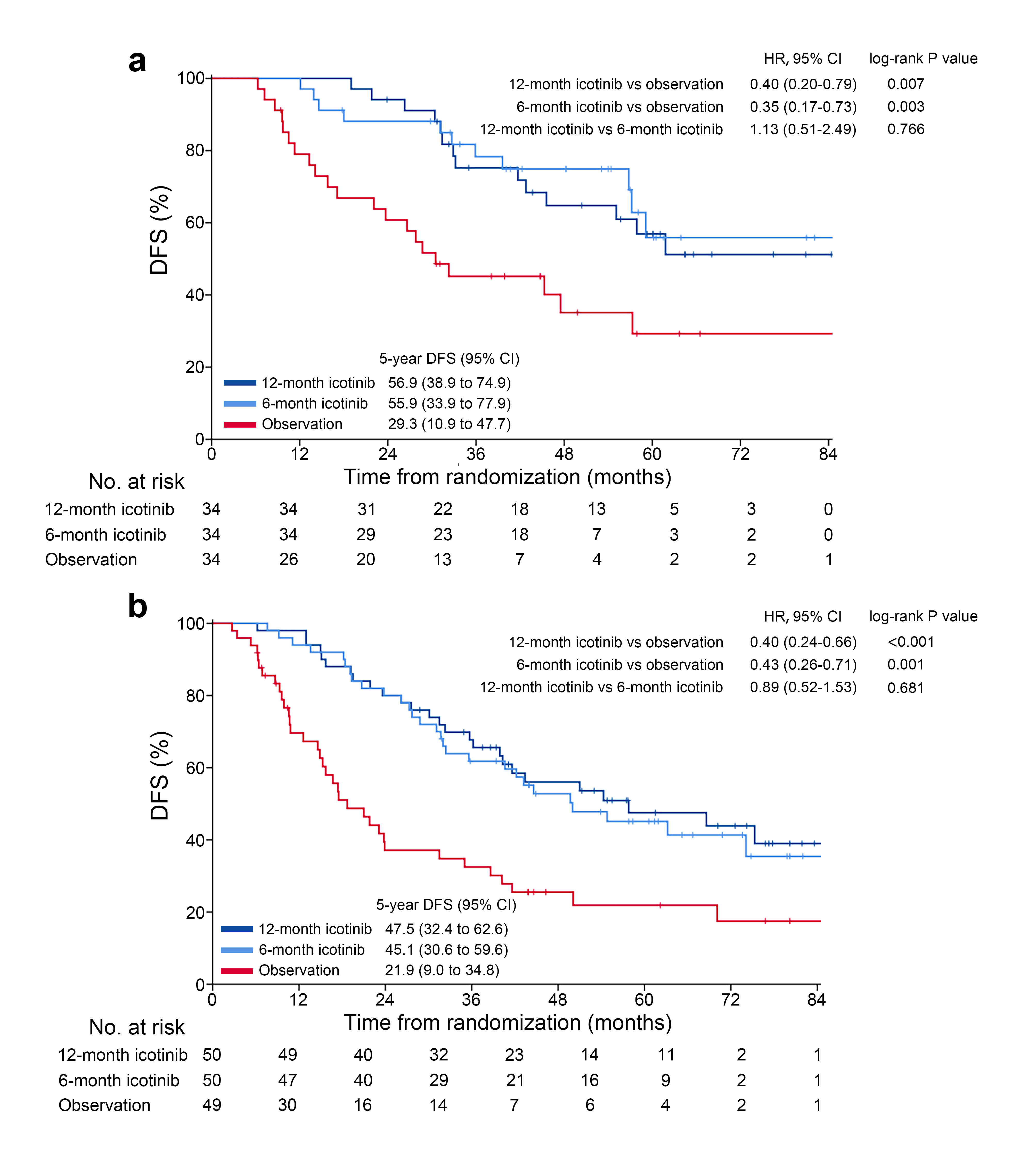


Supplementary Figure 2.

DFS in the subgroup of patients with stage II or IIIA NSCLC. (a) Kaplan-Meier estimates of DFS for patients with stage II disease. (b) Kaplan-Meier estimates of DFS for patients with stage IIIA disease. DFS, disease-free survival; NSCLC, non-small-cell lung cancer; HR, hazard ratio; CI, confidence interval.


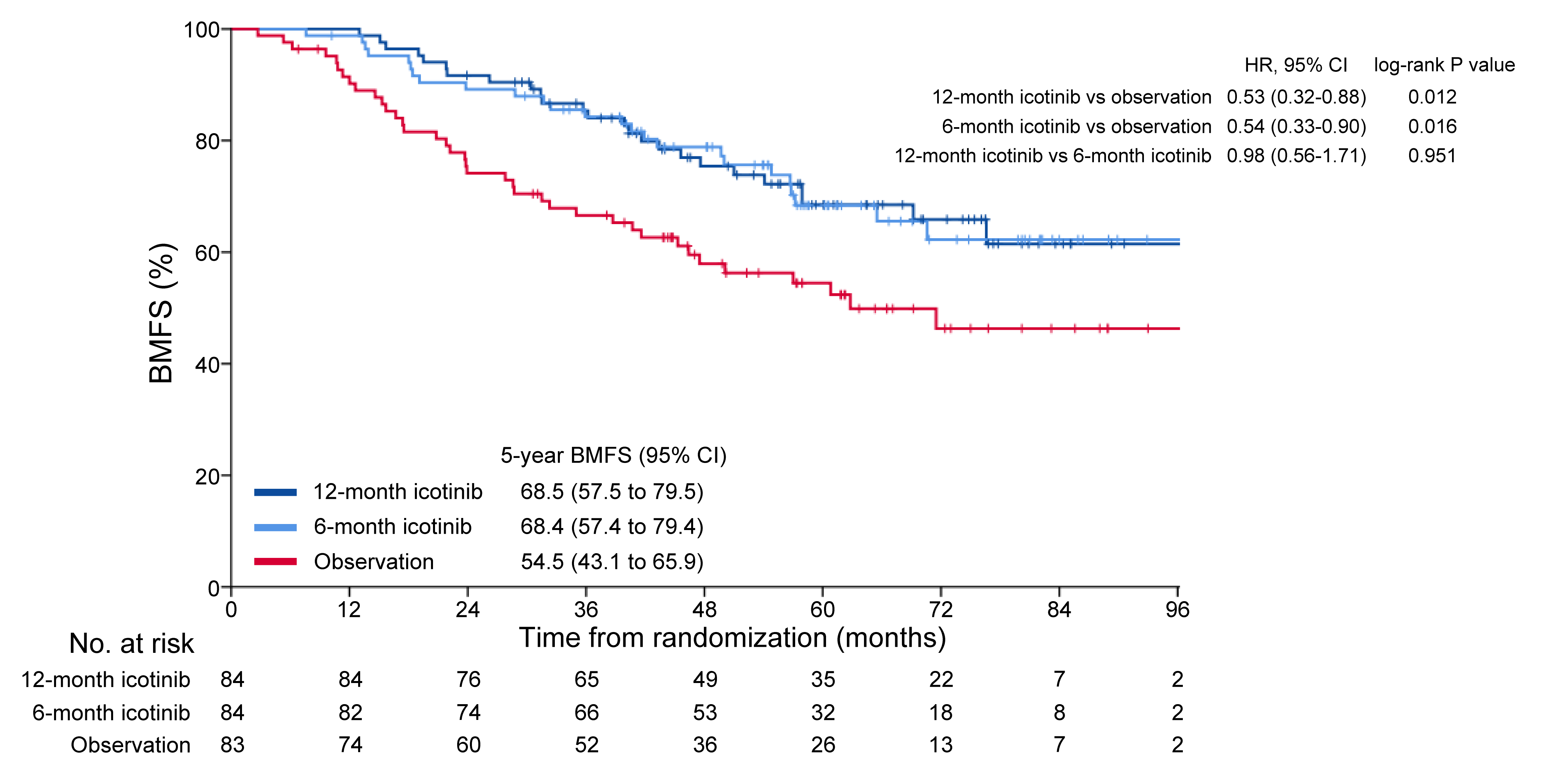


Supplementary Figure 3.

Kaplan-Meier estimates of BMFS by the investigator in the ITT population. BMFS, brain-metastasis-free survival; HR, hazard ratio; CI, confidence interval; ITT, intention-to-treat.


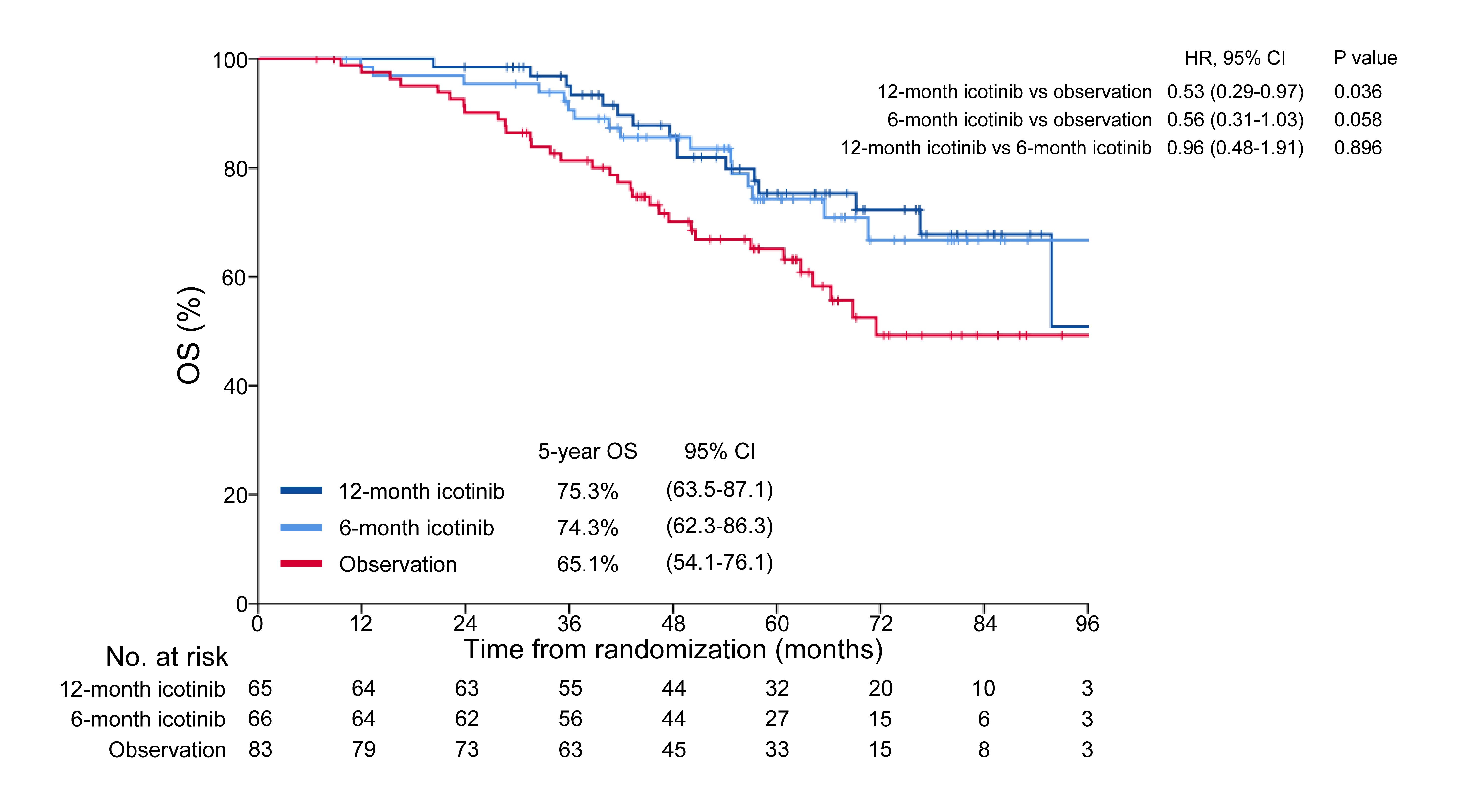


Supplementary Figure 4.

Kaplan-Meier estimates of OS in the per-protocol population. OS, overall survival; HR, hazard ratio; CI, confidence interval.


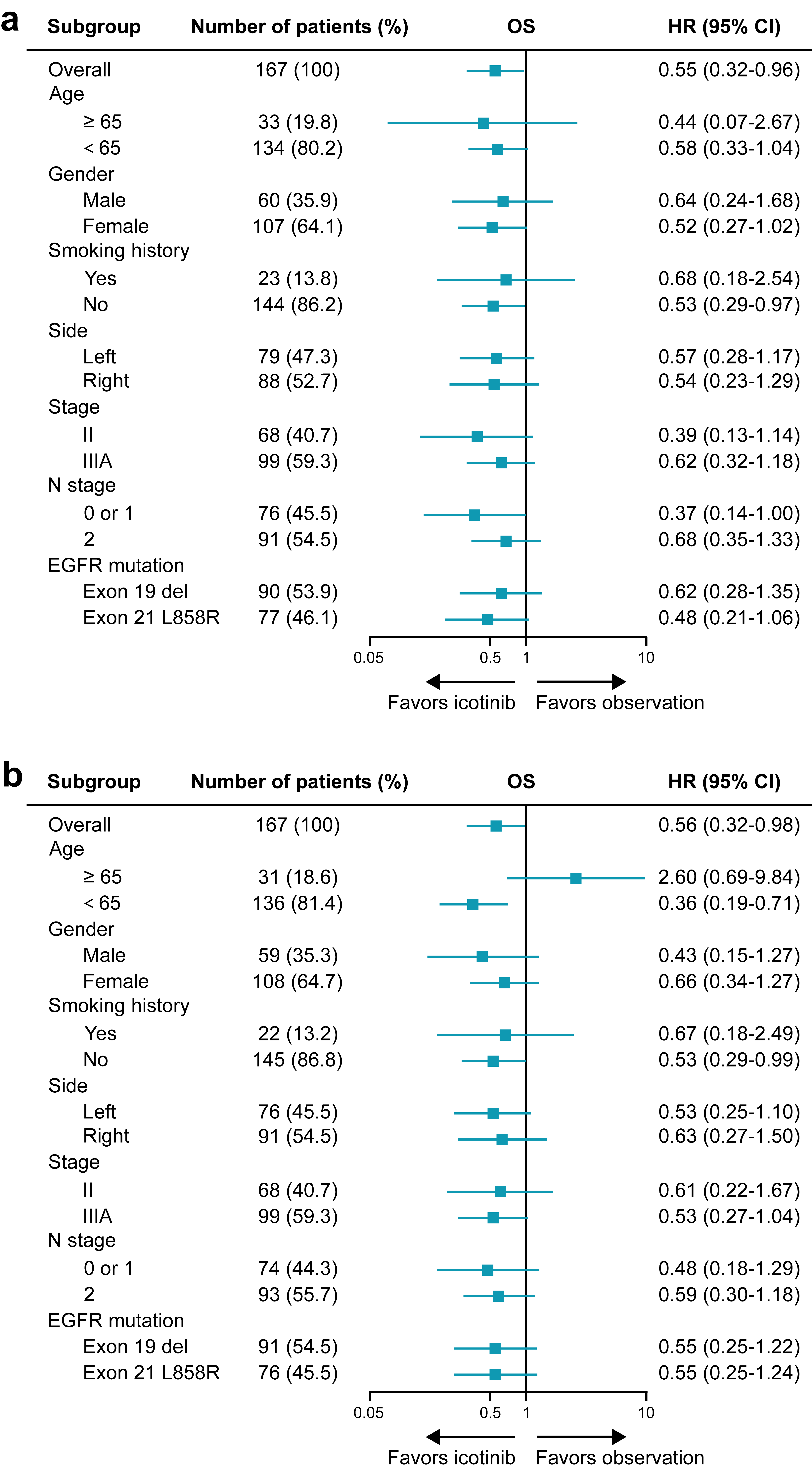


Supplementary Figure 5.

Forest plots of OS by subgroup.

(a) Subgroup analyses of OS for 12-month icotinib versus observation. (b) Subgroup analyses of OS for 6-month icotinib versus observation. Subgroups were either pre-planned (sex, stage, and EGFR mutation type) or post hoc (age, smoking history, N stage, and side). OS, overall survival; HR, hazard ratio; CI, confidence interval; EGFR, epidermal growth factor receptor.

Supplementary Table 1.

Adjuvant chemotherapy cycles and regimens.

|  | Icotinib for 12 months (n=84) | Icotinib for 6 months (n=84) | Observation (n=83) |
| --- | --- | --- | --- |
| Chemotherapy cycles |  |  |  |
| Four | 68 (81.0) | 66 (78.6) | 67 (80.7) |
| Three | 6 (7.1) | 7 (8.3) | 7 (8.4) |
| Two | 10 (11.9) | 11 (13.1) | 9 (10.8) |
| Chemotherapy regimens |  |  |  |
| Pemetrexed/carboplatin | 59 (70.2) | 60 (71.4) | 57 (68.7) |
| Vinorelbine/carboplatin | 18 (21.4) | 18 (21.4) | 19 (22.9) |
| Gemcitabine/carboplatin | 5 (6.0) | 5 (6.0) | 4 (4.8) |
| Docetaxel/carboplatin | 2 (2.4) | 1 (1.2) | 3 (3.6) |

Data are presented as n (%).

Supplementary Table 2.

Treatment effect of 12-month icotinib over 6-month icotinib on DFS in subgroup analyses.

| Variables | Subgroup | N (%) | HR (95% CI) | P value |
| --- | --- | --- | --- | --- |
| Total |  | 168 | 0.97 (0.62-1.51) | 0.887 |
| Age | ≥65 | 34 (20.2) | 0.43 (0.15-1.17) | 0.098 |
|  | ＜65 | 134 (79.8) | 1.22 (0.74-2.00) | 0.434 |
| Gender | Male | 59 (35.1) | 0.75 (0.36-1.55) | 0.436 |
|  | Female | 109 (64.9) | 1.12 (0.64-1.95) | 0.701 |
| Smoking history | Yes | 23 (13.7) | 0.74 (0.29-1.93) | 0.538 |
|  | No | 145 (86.3) | 1.03 (0.63-1.70) | 0.898 |
| Side | Left | 75 (44.6) | 0.68 (0.37-1.25) | 0.214 |
|  | Right | 93 (55.4) | 1.41 (0.73-2.75) | 0.309 |
| Stage | II | 68 (40.5) | 1.13 (0.51-2.49) | 0.766 |
|  | IIIA | 100 (59.5) | 0.89 (0.52-1.53) | 0.681 |
| N stage | 0 or 1 | 76 (45.2) | 1.14 (0.54-2.42) | 0.734 |
|  | 2 | 92 (54.8) | 0.89 (0.52-1.55) | 0.686 |
| EGFR mutation | Exon 19 del | 93 (55.4) | 0.81 (0.44-1.50) | 0.502 |
|  | Exon 21 L858R | 75 (44.6) | 1.17 (0.61-2.24) | 0.629 |

Abbreviations: DFS, disease-free survival; N, number; HR, hazard ratio; CI, confidence interval; EGFR, epidermal growth factor receptor.

Supplementary Table 3.

Site(s) of first disease relapse.

| Site | Icotinib for 12 months (n=84) | Icotinib for 6 months (n=84) | Observation (n=83) |
| --- | --- | --- | --- |
| Lung | 11 (13.1) | 12 (14.3) | 20 (24.1) |
| Brain | 10 (11.9) | 12 (14.3) | 15 (18.1) |
| Lymph nodes | 7 (8.3) | 6 (7.1) | 12 (14.5) |
| Bone | 5 (6.0) | 4 (4.8) | 8 (9.6) |
| Pleura | 3 (3.6) | 2 (2.4) | 6 (7.2) |
| Liver | 1 (1.2) | 1 (1.2) | 3 (3.6) |
| Adrenal | 1 (1.2) | 0 | 1 (1.2) |

Data are presented as n (%). Some patients had more than one site of relapse.

Supplementary Table 4.

Types of first disease relapse.

| Type | Icotinib for 12 months (n=84) | Icotinib for 6 months (n=84) | Observation  (n=83) |
| --- | --- | --- | --- |
| Relapse without death | 30 (35.7) | 30 (35.7) | 45 (54.2) |
| Local only | 16 (19.0) | 16 (19.0) | 22 (26.5) |
| Distant only | 11 (13.1) | 10 (11.9) | 17 (20.5) |
| Local and distant | 3 (3.6) | 4 (4.8) | 6 (7.2) |

Data are presented as n (%).

Supplementary Table 5.

Summary of the first subsequent therapy.

| Subsequent therapy | Icotinib for 12 months | Icotinib for 6 months | Observation |
| --- | --- | --- | --- |
| Patients who experienced a DFS event | 40 | 39 | 56 |
| EGFR-TKI |  |  |  |
| Osimertinib | 9 (22.5) | 8 (20.5) | 5 (8.9) |
| Other 3rd-generation | 8 (20.0) | 4 (10.3) | 4 (7.1) |
| Afatinib | 1 (2.5) | 4 (10.3) | 3 (5.4) |
| Icotinib | 2 (5.0) | 4 (10.3) | 24 (42.9) |
| Other 1st-generation | 3 (7.5) | 6 (15.4) | 7 (12.5) |
| Chemotherapy | 4 (10.0) | 3 (7.7) | 1 (1.8) |
| Apatinib | 1 (2.5) | 1 (2.6) | 0 |
| Anlotinib | 0 | 2 (5.1) | 1 (1.8) |
| Crizotinib | 1 (2.5) | 0 | 0 |
| Bevacizumab | 1 (2.5) | 0 | 0 |
| PD-1/PD-L1 inhibitors | 0 | 1 (2.6) | 0 |
| Radiotherapy | 3 (7.5) | 3 (7.7) | 1 (1.8) |
| No subsequent therapy^a^ | 11 (27.5) | 10 (25.6) | 12 (21.4) |

The data are n (%). Percentages of patients are calculated from the number of patients who experienced a DFS event. Some patients had more than one first subsequent therapy.

^a^Including patients who had died without relapse.

Abbreviations: DFS, disease-free survival; EGFR, epidermal growth factor receptor; TKI, tyrosine kinase inhibitor; PD-1, programmed cell death protein-1; PD-L1, programmed cell death-ligand 1.

Supplementary Table 6.

Summary of the subsequent therapies.

| Subsequent therapy | Icotinib for 12 months | Icotinib for 6 months | Observation |
| --- | --- | --- | --- |
| Patients who experienced a DFS event | 40 | 39 | 56 |
| EGFR-TKI |  |  |  |
| Osimertinib | 14 (35.0) | 13 (33.3) | 18 (32.1) |
| Other 3rd-generation | 11 (27.5) | 12 (30.8) | 14 (25.0) |
| Afatinib | 1 (2.5) | 4 (10.3) | 4 (7.1) |
| Icotinib | 4 (10.0) | 7 (17.9) | 27 (48.2) |
| Other 1st-generation | 3 (7.5) | 6 (15.4) | 8 (14.3) |
| Chemotherapy | 6 (15.0) | 6 (15.4) | 3 (5.4) |
| Apatinib | 3 (7.5) | 3 (7.7) | 3 (5.4) |
| Anlotinib | 3 (7.5) | 6 (15.4) | 5 (8.9) |
| Crizotinib | 2 (5.0) | 1 (2.6) | 2 (3.6) |
| Bevacizumab | 3 (7.5) | 5 (12.8) | 2 (3.6) |
| PD-1/PD-L1 inhibitors | 0 | 4 (10.3) | 1 (1.8) |
| Cetuximab | 0 | 1 (2.6) | 0 |
| Radiotherapy | 5 (12.5) | 4 (10.3) | 3 (5.4) |
| No subsequent therapy^a^ | 11 (27.5) | 10 (25.6) | 12 (21.4) |

The data are n (%). Percentages of patients are calculated from the number of patients who experienced a DFS event. Some patients had multiple subsequent therapies.

^a^Including patients who had died without relapse.

Abbreviations: DFS, disease-free survival; EGFR, epidermal growth factor receptor; TKI, tyrosine kinase inhibitor; PD-1, programmed cell death protein-1; PD-L1, programmed cell death-ligand 1.

Supplementary Table 7.

Summary of adverse events.

| AE | Icotinib for 12 months  (n=84) | Icotinib for 6 months (n=84) | Observation (n=83) |
| --- | --- | --- | --- |
| Any AE | 65 (77.4) | 62 (73.8) | 35 (42.2) |
| Any AE ≥ grade 3 | 7 (8.3) | 5 (6.0) | 2 (2.4) |
| Any AE leading to treatment discontinuation | 4 (4.8) | 3 (3.6) | 0 |
| Any AE leading to dose reduction | 0 | 0 | 0 |
| ILD | 0 | 0 | 0 |
| Fatal AE | 0 | 0 | 0 |

Data are presented as n (%). All adverse events were assessed according to the National Cancer Institute Common Terminology Criteria for Adverse Events, version 4.0.

Abbreviations: AE, adverse event; ILD, interstitial lung disease.
