# Supplementary material for: Adjuvant icotinib for resected EGFR-mutated stage II–IIIA non-small-cell lung cancer (ICTAN, GASTO1002): a randomized comparison study
Source: Signal Transduct Target Ther. 2025 Aug 28;10:273. doi: 10.1038/s41392-025-02358-w (PMC12391381; doi:10.1038/s41392-025-02358-w)
Supplement: Supplementary file 2 — Trial Protocol [file 41392_2025_2358_MOESM2_ESM.docx]

**Clinical Trial Protocol**

Adjuvant icotinib of 12 months or 6 months versus observation following adjuvant chemotherapy for resected EGFR-mutant stage II–IIIA non-small-cell lung cancer (ICTAN, GASTO1002): a randomized phase 3 trial

Principal Investigator: Si-Yu Wang

Clinical Research Department: Thoracic Surgery

Sponsoring Company: Betta Pharmaceuticals Co., Ltd.

Protocol Number: GASTO1002

Registration No.: NCT01996098

Protocol Version Number: 3.0, September 1, 2020

Confidentiality Statement

The information contained in this document (especially unpublished data) is the property of the sponsor. Therefore, the information provided to you (about investigators, potential investigators, or consultants) is confidential and may only be used for review by you, your employees, or the relevant ethics committee. Except in situations where information needs to be provided to obtain informed consent from potential patients, the information in this document must not be disclosed to others without the written permission of the sponsor.

PROTOCOL SYNOPSIS

| **Title** | Adjuvant icotinib of 12 months or 6 months versus observation following adjuvant chemotherapy for resected EGFR-mutant stage II–IIIA non-small-cell lung cancer (ICTAN, GASTO1002): a randomized phase 3 trial |
| --- | --- |
| **Principal Investigator** | Si-Yu Wang |
| **Objective** | To further investigate the efficacy of sequential chemotherapy and an epidermal growth factor receptor tyrosine kinase inhibitor (EGFR-TKI) as postoperative adjuvant therapy for EGFR-mutant non-small-cell lung cancer; To explore the optimal duration of EGFR-TKI use in the adjuvant setting. |
| **Endpoints** | Primary Endpoint: disease-free survival (DFS) according to investigator assessment.  Secondary Endpoints: overall survival (OS), brain-metastasis-free survival (BMFS), safety and tolerability, and quality of life (QoL). |
| **Study Design** | Multi-center, randomized, prospective, open-label, phase III trial |
| **Number of cases** | 318 |
| **Target population** | Postoperative stage II-IIIA (7th edition of TNM staging) EGFR-mutant non-small-cell lung cancer patients who have received 2-4 cycles of adjuvant chemotherapy. |
| **Inclusion Criteria** | **Inclusion Criteria Related to the Disease:**  **•** Postoperative histopathology confirmed R0 resection for stage II-IIIA non-small-cell lung cancer, accompanied by centrally confirmed EGFR mutation positivity (exon 19 deletion or exon 21 L858R mutation);  • Received 2-4 cycles of adjuvant chemotherapy postoperatively; the adjuvant chemotherapy regimen consists of third-generation chemotherapy drugs (gemcitabine, vinorelbine, paclitaxel, or docetaxel) combined with carboplatin or cisplatin. For patients with lung adenocarcinoma, pemetrexed combined with carboplatin or cisplatin can be used;  • Have not received any other chemotherapy, radiotherapy, or biotherapy except for the 2-4 cycles of adjuvant chemotherapy;  • Treatment initiation within 4 weeks after the completion of 2-4 cycles of adjuvant chemotherapy;  • No signs of tumor recurrence in pre-treatment examinations.  **Hematological, Biochemical, and Organ Function:**  • Hemoglobin ≥ 100 g/L (can be maintained or exceeded this level by transfusion);  • Absolute neutrophil count ≥ 2.0 × 10^9^/L;  • Platelet count ≥ 100 × 10^9^/L;  • Total bilirubin ≤ 1.5 times the upper limit of normal;  • Alanine aminotransferase and aspartate aminotransferase ≤ 2.5 times the upper limit of normal;  • Creatinine ≤ 1.25 times the upper limit of normal; and creatinine clearance rate ≥ 60 mL/min;  • Women of childbearing age (15-49 years) should have a negative urine pregnancy test within 7 days.  **General Inclusion Criteria:**  **•** Obtained informed consent signed by the patient or their legal representative;  • Patient compliance is good;  • Able to take oral medication;  • Male or female aged 18-75 years;  • ECOG performance status score of 0-1;  • Life expectancy greater than 12 weeks;  • Male and female patients of childbearing age agree to use reliable contraceptive methods before entering the trial, during the study, and until 8 weeks after discontinuation of the drug. |

| **Exclusion Criteria** | • Received any systemic anticancer therapy other than adjuvant chemotherapy for NSCLC, including cytotoxic drug therapy, targeted drug therapy (including tyrosine kinase inhibitors or monoclonal antibodies), experimental therapy;  • Received local radiotherapy for NSCLC;  • Have physiological imperfections in the upper gastrointestinal tract, or malabsorption syndrome, or inability to tolerate oral medication, or active gastrointestinal ulcers;  • Have clinical objective evidence (pathological or imaging) confirming tumor recurrence before the start of treatment;  • Patients who have had other cancers within five years prior to the start of treatment in this study, excluding cervical carcinoma in situ, cured basal cell carcinoma, bladder epithelial tumors [including Ta and Tis];  • Known allergy to icotinib or any component of this product;  • History of interstitial lung disease, drug-induced interstitial lung disease, or any clinically evident active interstitial lung disease;  • Idiopathic pulmonary fibrosis found on baseline CT scan;  • Uncontrolled ocular inflammation or infection, or any condition that may lead to the aforementioned eye diseases;  • Any unstable systemic disease, including: active infection, uncontrolled hypertension, unstable angina, angina pectoris with onset within the last 3 months, congestive heart failure (≥ New York Heart Association [NYHA] II level), myocardial infarction (within 6 months before enrollment), severe arrhythmia requiring medication, liver, kidney or metabolic diseases;  • Known human immunodeficiency virus (HIV) infection;  • Patients with mixed small cell lung cancer components;  • Pregnant or breastfeeding women;  • History of definite neurological or psychiatric disorders, including epilepsy or dementia;  • Other conditions deemed unsuitable for enrollment by the investigator. |
| --- | --- |
| **Study duration** | Based on an estimated enrollment of about 5 patients per month, a total of 318 patients are planned to be enrolled, which will take approximately 64 months. The minimum survival follow-up period is 5 years, making the total study duration approximately 124 months. |
| **Study groups** | **Group A (12-month icotinib group):**  After R0 resection and 2-4 cycles of adjuvant chemotherapy, patients began to receive oral administration of icotinib 125mg, tid, for a duration of 12 months, or discontinued treatment due to tumor recurrence or intolerance.  **Group B (6-month icotinib group):**  After R0 resection and 2-4 cycles of adjuvant chemotherapy, patients began to receive oral administration of icotinib 125mg, tid, for a duration of 6 months, or discontinued treatment due to tumor recurrence or intolerance.  **Group C (Observation group):**  After R0 resection and 2-4 cycles of adjuvant chemotherapy, patients underwent observation. |
| Treatment period & Follow-up period | After patients sign the informed consent form and meet the inclusion and exclusion criteria, they are randomly assigned to three groups: the 12-month icotinib group, the 6-month icotinib group, and the observation group. The planned sample size is 106 in each group.  Patients are scheduled for visits of assessment once a month during the medication period. Follow-up assessments were scheduled every 3 months for the first 2 years after randomization, every 6 months until 5 years, and every 12 months thereafter until disease relapse or death.  After disease progression, researchers need to contact the patients, their families, or their current physicians by phone at least once every 3 months to collect long-term follow-up information on survival. Patients who withdraw from the study treatment for reasons other than progression (excluding patients who withdraw consent, are lost to follow-up, or die) should continue to receive objective tumor assessments every 3 months to collect information about disease progression. |
| Safety | All patients who have received icotinib and those in the observation group will be included in the safety analysis population. The physical examination results, vital signs, adverse events, and abnormal laboratory test values of the patients will be summarized. Adverse events should be reported and graded according to the National Cancer Institute's Common Terminology Criteria for Adverse Events (CTCAE) version 4.0. |
| Sample size | Statistical hypothesis and sample size calculation: A sample size of 318 (requiring 198 events) is based on the following considerations: From previous studies, it is assumed that the median DFS is 30 months for patients with EGFR-mutated stage II-IIIA NSCLC following adjuvant chemotherapy, with a hazard ratio (HR) of 0.6, a power of test (1-β) of 85%, a significance level (α) of 5% for a two-sided test, and an overall dropout rate of 5%. |

# STUDY OVERVIEW

**Primary endpoint：**

DFS according to investigator assessment.

**Secondary endpoints：**

OS

BMFS

Safety

QoL

Patients with postoperative pathological confirmed stage IIA-IIIA EGFR mutation-positive NSCLC who had undergone R0 resection and adjuvant chemotherapy for 2-4 cycles

Follow-up

**Icotinib for 12 months group**

(N=106)

**Icotinib for 6 months group**

(N=106)

**Observation group**

(N=106)

# Table 1. Study Plan

| **Study Plan** | **Study items** | **Screening** | | **Treatment Period** | | | | | **Post- Treatment^M^** |
| --- | --- | --- | --- | --- | --- | --- | --- | --- | --- |
|  |  |  |  | **Group A** | | **Group B** | | **Group C** |  |
|  |  | **Screening Period** | | **C1-C12**  Every 30 days | **Follow-up**  Every 90 days | **C1-C6**  Every 30 days | **Follow-up**  Every 90 days | **Follow-up**  Every 90 days | **Follow up**  **for survival** |
|  |  | **Day -28 to Day 0** | **Day -7 to Day 0** | **Day 1** |  | **Day 1** |  |  |  |
| **Screening stage** | Written informed consent /demography /medical history **^A^**/previous treatment records | 🗙 |  |  |  |  |  |  |  |
|  | Inclusion/exclusion criteria |  | 🗙 |  |  |  |  |  |  |
|  | Urine pregnancy test **^B^** |  | 🗙 |  |  |  |  |  |  |
|  | Pulmonary function test/HIV antibody |  | 🗙 |  |  |  |  |  |  |
| **Safety data** | ECG **^C^** |  | 🗙 |  |  |  |  |  |  |
|  | ECOG performance status/height/weight/vital signs **^D^** |  | 🗙 | 🗙 | 🗙 | 🗙 | 🗙 | 🗙 |  |
|  | Adverse event **^E^** |  | 🗙 | 🗙 |  | 🗙 |  | 🗙 |  |
|  | Drug usage record **^F^** |  |  | 🗙 |  | 🗙 |  | 🗙 |  |
| **Follow-up examination** | Physical examination |  | 🗙 | 🗙 | 🗙 | 🗙 | 🗙 | 🗙 |  |
|  | Chest+upper abdominal enhanced CT **^G^** | 🗙 |  |  | 🗙 |  | 🗙 | 🗙 |  |
|  | Assessment of other body parts **^H^** | 🗙 |  |  |  |  |  |  |  |
|  | Hematology/biochemistry/urinalysis **^I^** |  | 🗙 | 🗙 | 🗙 | 🗙 | 🗙 | 🗙 |  |
|  | FACT-L and LCSS questionnaires **^J^** |  | 🗙 | 🗙 | 🗙 | 🗙 | 🗙 | 🗙 |  |
|  | Follow-up treatment and survival status |  |  |  |  |  |  |  | 🗙 |
| **Exploratory analyses** | Tissue specimens or sections for biomarker analysis **^K^** | 🗙 |  |  |  |  |  |  |  |
|  | Blood for biomarker analysis (optional) **^L^** |  | 🗙 | 🗙 | 🗙 | 🗙 | 🗙 | 🗙 |  |

1. Complete medical history (including a complete history of internal and surgical diseases), demographics, past surgical history, concomitant medications, comorbidities, allergy history, and smoking history. Special attention to past and present lung diseases and/or systemic diseases involving the lungs (such as connective tissue diseases). Smoking status was only inquired about at screening.
2. Premenopausal women of childbearing potential should be negative. If a suspicious pregnancy event occurs during the trial, a pregnancy test must be repeated, and if positive, it must be reported immediately.
3. If there are electrocardiographic abnormalities and clinical symptoms, LVEF should be checked.
4. Vital signs include heart rate, blood pressure, respiratory rate, and temperature.
5. If any subjective ocular symptoms or new or worsening respiratory symptoms (such as coughing, wheezing) occur, patients should be given appropriate medical assistance immediately. Any symptoms should be treated according to clinical routine, and if they meet the definition, they should also be reported as adverse events or serious adverse events (SAEs). The collection of adverse events starts from the patient signing the informed consent form. After the end of the study, all unresolved adverse events or serious adverse events must be followed up until resolution, unless the investigator believes that they are unlikely to resolve due to the patient's own disease. All new adverse events and serious adverse events occurring within 30 days after the last dose of the study drug must be reported (if serious adverse events, they must be reported to the ethics committee of the study center, Zhejiang Betta Pharmaceuticals Co., Ltd., and the National Medical Products Administration within 24 hours) and followed up as mentioned above until resolution. Record the most severe level of adverse events for each cycle in the CRF.
6. Collect data on concomitant medications, including drug dosage, route of administration, dosing schedule, start date, indication, end date, and record from the random use of study medication until one month after discontinuation. The physician obtains this information from the patient's follow-up every 30 days. By the drugs routinely prescribed for patients, the physician will evaluate whether changes in concomitant medications over the past 30 days may lead to a clinically significant improvement in pulmonary symptoms.
7. Pretreatment and evaluation of tumor recurrence imaging examinations must include chest and upper abdominal enhanced CT. Tumor assessment is conducted once every 3 months, with a window period of +/- 7 days, covering the chest and upper abdomen. If new lesions are suspected in any area, CT scans should be performed until disease recurrence. All imaging examinations can be replaced by PET/CT.
8. Brain MRI and bone scans were scheduled every 12 months or performed as indicated based on symptoms.
9. Hematology (hemoglobin, platelets, white blood cells, absolute neutrophil count, absolute lymphocyte count, etc.)/blood biochemistry (blood glucose, calcium, phosphorus, sodium, potassium, chlorine, creatinine, blood urea nitrogen, total protein, albumin, alanine aminotransferase, aspartate aminotransferase, alkaline phosphatase, total bilirubin, etc.)/urinalysis (urine pH value, urine sugar, urine protein, and occult blood, etc.) should be completed within 7 days before the first dose, at each follow-up, and within 7 days before and after exiting the study, as well as when clinically indicated, and the number of times can be increased according to the actual situation.
10. The patient self-assessment questionnaire FACT-L and LCSS should be completed at each visit of assessment.
11. Applicable for patients from whom tissue specimens can be obtained. Paraffin blocks or paraffin sections (slices of 5 micrometer) of tumor lesions will be collected from patients entering the study.
12. A 10ml whole blood sample for biomarker analysis should be taken before treatment and at each visit.
13. The follow-up time window after recurrence is every 90 days ± 7 days.

**Table 2. List of investigators**

| **Site** | **Investigator** |
| --- | --- |
| Sun Yat-sen University Cancer Center | Si-Yu Wang (PI), Hao Long (co-PI), Ning Li, Wei Ou |
| The First Affiliated Hospital of Sun Yat-sen University | Chao Cheng (PI), Weixiong Yang |
| Tianjin Medical University Cancer Institute and Hospital | Jian You (PI), Feng Xu |
| Shenzhen People’s Hospital | Lin Yang (PI), Guanggui Ding |
| Hainan General Hospital | Feng-Xia Chen (PI), Xian-Shan Chen |
| Zhongshan City People's Hospital | Yi Liang (PI), Ronggui Hu |
| Affiliated Hospital of Guangdong Medical University | Zhixiong Yang (PI), Shujun Li |

**TABLE OF CONTENTS PAGE**

[PROTOCOL SYNOPSIS 2](#_Toc170380163)

[STUDY OVERVIEW 6](#_Toc170380164)

[Table 1. Study Plan 7](#_Toc170380165)

[Table 2. List of investigators 9](#_Toc170380165)

[1. INTRODUCTION 13](#_Toc170380166)

[2. STUDY OBJECTIVES 15](#_Toc170380168)

[2.1. Primary objective 15](#_Toc170380169)

[2.2. Secondary objectives 15](#_Toc170380170)

[2.3. Exploratory objectives 15](#_Toc170380171)

[3. STUDY DESIGN 15](#_Toc170380172)

[3.1. Study design and plan 15](#_Toc170380173)

[3.2. Subject selection 16](#_Toc170380174)

[3.2.1. Inclusion criteria 16](#_Toc170380175)

[3.2.2. Exclusion criteria 17](#_Toc170380176)

[3.2.3. Definition of R0 resection 17](#_Toc170380176)

[3.3. Study duration 18](#_Toc170380177)

[3.4. Withdrawal from study 18](#_Toc170380178)

[3.4.1. Withdrawal criteria 18](#_Toc170380179)

[3.5. Study plan 19](#_Toc170380180)

[3.5.1. Methods for assigning treatment groups 19](#_Toc170380181)

[3.5.2. Withdrawal of icotinib 20](#_Toc170380182)

[3.5.3. Combined therapy and smoking 20](#_Toc170380183)

[3.5.4. Concomitant medication 20](#_Toc170380184)

[3.5.5. Compliance 21](#_Toc170380185)

[3.6. Study endpoint 21](#_Toc170380186)

[3.6.1. Efficacy endpoint 21](#_Toc170380187)

[3.6.2. Security specifications 22](#_Toc170380188)

[3.7. Study process (See Table 1) 22](#_Toc170380189)

[3.7.1. Screening period 22](#_Toc170380190)

[3.7.2. Treatment period 23](#_Toc170380191)

[3.7.3. Survival follow-up 24](#_Toc170380192)

[3.7.4. Temporary visit 24](#_Toc170380193)

[3.8. Quality of data 24](#_Toc170380194)

[3.9. Archiving 24](#_Toc170380195)

[4. INVESTIGATIONAL DRUG 25](#_Toc170380196)

[4.1. Icotinib 25](#_Toc170380197)

[4.1.1. Clinical trial drug name, physicochemical properties, appearance, specifications, composition, usage, storage 25](#_Toc170380198)

[4.1.2. Drug packaging and labeling 26](#_Toc170380199)

[4.1.3. Drug distribution and inventory 26](#_Toc170380200)

[5. ETHICAL AND LEGAL ASPECTS 26](#_Toc170380201)

[5.1. Independent ethics committee (IEC) 26](#_Toc170380202)

[5.2. Ethical guidelines for this study 27](#_Toc170380203)

[5.3. Patient information and informed consent 27](#_Toc170380204)

[5.4. Confidentiality 27](#_Toc170380205)

[5.5. Conditions for amending the study protocol 28](#_Toc170380206)

[5.6. Conditions for terminating the study 28](#_Toc170380207)

[5.7. Preservation of study documents, case report forms, and records 28](#_Toc170380208)

[5.7.1. Preservation of investigator documents 28](#_Toc170380209)

[5.7.2. Original records and background data information 29](#_Toc170380210)

[5.7.3. Audit and inspection 29](#_Toc170380211)

[5.7.4. Case report forms 29](#_Toc170380212)

[6. MONITORING OF THE STUDY 29](#_Toc170380213)

[7. PUBLICATION OF RESEARCH DATA AND PROTECTION OF TRADE SECRETS 30](#_Toc170380214)

[8. STATISTICAL AND ANALYSIS PLAN 30](#_Toc170380215)

[8.1. Primary and secondary variables 30](#_Toc170380216)

[8.1.1. Primary variables 30](#_Toc170380217)

[8.1.2. Secondary variable 30](#_Toc170380218)

[8.2. Statistical analysis 31](#_Toc170380219)

[8.2.1. Baseline and demographic characteristics 31](#_Toc170380220)

[8.2.2. Primary efficacy 31](#_Toc170380221)

[8.2.3. Secondary efficacy 31](#_Toc170380222)

[8.2.4. Exploratory analysis 31](#_Toc170380223)

[8.3. Type of analysis 31](#_Toc170380224)

[8.4. Interim analysis 32](#_Toc170380225)

[8.5. Safety analysis 32](#_Toc170380226)

[9. PRINCIPLE OF SAFETY 32](#_Toc170380227)

[9.1. Precautions/Warnings 32](#_Toc170380228)

[9.2. Adverse events 33](#_Toc170380229)

[9.2.1. Definition of adverse events 33](#_Toc170380230)

[9.2.2. Adverse event grading 34](#_Toc170380231)

[9.2.3. Monitoring of adverse events 34](#_Toc170380232)

[9.2.4. Relationship between adverse events and the study drug 34](#_Toc170380233)

[9.2.5. Definitions of serious adverse events 35](#_Toc170380234)

[9.2.6. Malignant tumor progression 36](#_Toc170380235)

[9.2.7. Unexpected adverse events 37](#_Toc170380236)

[9.2.8. Reporting of adverse events 37](#_Toc170380237)

[9.2.9. Treatment and follow-up of adverse events 38](#_Toc170380238)

[9.2.10. Laboratory test value abnormalities 38](#_Toc170380239)

[9.3. Pregnancy 39](#_Toc170380240)

[10. INDEPENDENT DATA MONITORING COMMITTEE (IDMC) 40](#_Toc170380241)

[11. REFERENCES 41](#_Toc170380242)

[Investigator's Statement 43](#_Toc170380243)

[Summary of changes to the protocol 44](#_Toc170380243)

# INTRODUCTION

Lung cancer is the most common cause of death from cancer. Non-small-cell lung cancer (NSCLC) represents approximately 80% to 85% of all lung cancers ^[1,2]^. In 2006, the incidence of malignant tumors in urban populations in China surpassed that of cardiovascular and cerebrovascular diseases, becoming the most common cause of death, with lung cancer being the most common among malignant tumors. Among the annual new cases of lung cancer, more than 30% patients present with stages II-IIIA, and 50% of them have ipsilateral mediastinal lymph node metastasis, with a 5-year survival rate of only 10-15%. The local recurrence rate for patients with stage II-IIIA is 80%, and the distant metastasis rate is 60% ^[3,4]^. Even in early-stage (stage I) patients, the 5-year survival rate is only 58-73%, indicating that these patients still have a certain risk of recurrence ^[5]^. Therefore, the combination of local tumor control and systemic treatment has become an important treatment modality for resectable NSCLC patients ^[6]^.

In 1995, the Non-Small Cell Lung Cancer Collaborative Group (NSCLCCG) published a large meta-analysis of adjuvant chemotherapy in NSCLC based on individual patient data, which collected information from 24 randomized controlled clinical trials comparing adjuvant chemotherapy with surgery alone from 1961 to 1991, involving 4767 patients. In the meta-analysis, all-stage (IB-IIIA) hazard ratios (HR) was 0.86, corresponding to an absolute benefit of chemotherapy on overall survival (OS) of 4-5% at 5 years ^[7]^. Although the difference was not statistically significant (P=0.08), it preliminarily established the status of platinum-based regimens in the adjuvant setting in NSCLC. Over the next decade, several randomized controlled clinical trials compared various cisplatin-based regimens of adjuvant chemotherapy with surgery alone. Since the 21st century, several large-scale clinical randomized controlled trials have been reported. Some of them ^[8-10]^ confirmed the overall survival benefit of adjuvant chemotherapy, while others ^[11,12]^ yielded negative results. In 2008, LACE conducted a meta-analysis involving 5 large-sample clinical trials ^[13]^, with a total of 4584 patients and a median follow-up of 5.2 years. The results indicated that patients derived benefits in disease-free survival (DFS) and OS from chemotherapy treatment, with a 5-year DFS rate increase of 5.8% and a 5-year OS rate increase of 5.4%. The meta-analysis also clarified the role of a 4-cycle platinum-based chemotherapy regimen in improving the 5-year survival rate of patients with completely resected stage II to IIIA NSCLC. It is worth mentioning the CALGB9633 study, which was a randomized controlled study of adjuvant chemotherapy using a paclitaxel plus carboplatin regimen, in which all the patients were stage IB (6th edition of TNM staging). The subgroup analysis confirmed that patients with tumors ≥ 4cm could benefit from adjuvant chemotherapy ^[14]^.

With the continuous development of clinical research and application, epidermal growth factor receptors-tyrosine kinase inhibitors (EGFR-TKIs) are becoming increasingly important in treating NSCLC. Data from multiple trials, including IPASS, OPTIMAL and EURTAC, showed that EGFR-TKIs led to significantly longer progression-free survival (PFS) compared with that for chemotherapy in patients with EGFR-mutant stage IIIB-IV NSCLC ^[15,16,17]^. Thus, EGFR-TKIs have become the standard first-line treatment regimen for this population. Targeted adjuvant therapy for NSCLC after surgery is currently a hot topic of research. The phase III randomized BR.19 trial explored the efficacy of gefitinib as adjuvant therapy in NSCLC patients with stage IB to IIIA disease ^[18]^. Patients were randomly assigned to receive gefitinib or a placebo for 2 years after surgery, with 503 patients enrolled from 2002 to 2005. The results showed that there was no DFS or OS benefit from gefitinib for completely resected NSCLC. However, this trial did not select patients based on EGFR mutations. Another ongoing RADIANT phase III clinical study included patients with stages I to IIIA who were EGFR positive (IHC or FISH) after surgery and received erlotinib treatment for 2 years, evaluating the role of erlotinib in adjuvant therapy, with placebos as the control group. The enrolled population was also not selected based on EGFR mutations ^[19]^. In 2011, MSKCC retrospectively analyzed 167 cases of stage I-III EGFR-mutated lung adenocarcinoma patients from 2002 to 2008, of which 56 cases used targeted therapy during the perioperative period. In the survival analysis comparison with patients who did not use targeted therapy, the patients received targeted therapy had a higher 2-year DFS rate (89% vs 70%), although the p value (0.06) did not show a significant statistical difference, but the trend of benefit in the adjuvant targeted therapy group had already appeared ^[20]^. In the phase II SELECT study, which was reported at the 2012 ASCO meeting, EGFR-mutated stage I-III NSCLC patients were given erlotinib for 2 years after receiving standard adjuvant chemotherapy or adjuvant radiotherapy. The study recruited 36 patients, with 11 patients taking medication for less than 2 years. After a median follow-up of 2.5 years, the 2-year DFS rate reached 94% ^[21]^.

At the 2013 American Society of Clinical Oncology (ASCO) meeting, Professor Si-Yu Wang reported the results of a phase II clinical trial comparing pemetrexed plus carboplatin followed by gefitinib with pemetrexed plus carboplatin chemotherapy alone in postoperative IIIA-N2 stage EGFR mutation-positive NSCLC. The results showed that chemotherapy followed by gefitinib significantly prolonged DFS (39.8 vs 27.0 months). However, in this study, the duration of gefitinib treatment was 6 months, and in the gefitinib group, patients who relapsed after discontinuation of the drug still had a 50% response when gefitinib was reused ^[22]^. Therefore, up to now, there is still no consensus on how long postoperative TKI treatment should be administered.

Icotinib (Conmana®) is a small-molecule targeted anti-cancer drug independently developed by China for the treatment of advanced NSCLC, belonging to the EGFR-TKIs class of drugs. Since obtaining the clinical approval from the National Food and Drug Administration in 2006, a series of clinical studies have been completed, including a phase I clinical study on safety, tolerance, and pharmacokinetics in non-small-cell lung cancer patients with three doses per day, suggesting good tolerance and determining the subsequent study dosage regimen as 125mg TID ^[23]^. The ICOGEN study, namely "Icotinib versus gefitinib in previously treated advanced non-small-cell lung cancer (ICOGEN): a randomised, double-blind phase 3 non-inferiority trial", enrolled 399 patients. The efficacy results indicate that icotinib was non-inferior to gefitinib in terms of PFS (4.6 months vs 3.4 months), OS (13.3 months vs 13.9 months), ORR (27.6% vs 27.2%), and DCR (75.4% vs 74.9%). In terms of safety, the incidence of adverse events in the icotinib group was 60.5%, significantly lower than the 70.4% in the gefitinib group, with a statistically significant difference. The incidence of rash was 40% and 49.2%, respectively; the incidence of diarrhea was 18.5% and 27.6%, respectively, with no interstitial lung disease occurring in both groups ^[24]^. Therefore, the results of the ICOGEN study indicate that icotinib is non-inferior to gefitinib in terms of efficacy, but safer than gefitinib.

Based on the above research background, we took patients with EGFR mutation-positive stage II-IIIA NSCLC who had undergone R0 resection and adjuvant chemotherapy for 2-4 cycles as the research subjects, and randomly assigned them to icotinib treatment for 12 months or 6 months compared with the control group, to observe the efficacy and safety of icotinib in these patients, and to answer the question of how long TKI treatment following postoperative chemotherapy should be administered.

# STUDY OBJECTIVES

To investigate the efficacy and safety of an EGFR-TKI following adjuvant chemotherapy in stage II-IIIA EGFR-mutant NSCLC. Explore the optimal duration of EGFR-TKI use as adjuvant therapy following chemotherapy.

## Primary objective

Disease-free survival (DFS) according to investigator assessment.

## Secondary objectives

Overall survival (OS), brain-metastasis-free survival (BMFS), safety and tolerability, and quality of life (QoL).

## Exploratory objectives

Biomarker exploration.

# STUDY DESIGN

## Study design and plan

This is a multicenter, randomized, prospective, open-label phase III clinical trial aimed at assessing the efficacy and safety of icotinib treatment after adjuvant chemotherapy in patients with stage II-IIIA EGFR-mutated NSCLC who have undergone R0 resection.

A sample size of 318 (requiring 198 events) is based on the following considerations: From previous studies,

it is assumed that the median DFS is 30 months for patients with EGFR-mutated stage II-IIIA NSCLC following adjuvant chemotherapy, with a hazard ratio (HR) of 0.6, a power of test (1-β) of 85%, a significance level (α) of 5% for a two-sided test, and an overall dropout rate of 5%.

These 318 patients are planned to be enrolled in the study, after adjuvant chemotherapy, randomly assigned into three groups: the 12-month icotinib group, the 6-month icotinib group, and the observation group (control group). Patients in the icotinib groups will receive icotinib 125 mg orally three times a day for a continuous 12 or 6 months, or until disease recurrence or intolerable toxicity.

## Subject selection

Each subject should meet all of the inclusion criteria and none of the exclusion criteria for this study. Under no circumstances can there be exceptions to this rule.

### Inclusion criteria

1. Good patient compliance;
2. Able to take oral medication;
3. Male or female aged 18-75 years;
4. ECOG performance status score of 0 to 1; Life expectancy greater than 12 weeks;
5. Postoperative histopathology confirmed R0 resection of stage II-IIIA NSCLC, accompanied by centrally confirmed EGFR mutation positivity (exon 19 or 21);
6. Received 2-4 cycles of adjuvant chemotherapy postoperatively; the adjuvant chemotherapy regimen consists of third-generation chemotherapy drugs (gemcitabine, vinorelbine, paclitaxel, or docetaxel) combined with carboplatin or cisplatin; for patients with lung adenocarcinoma, pemetrexed combined with carboplatin or cisplatin can be used;
7. Never received other chemotherapy, radiation therapy, or biotherapy except for the 2-4 cycles of adjuvant chemotherapy;
8. Treatment starts within 4 weeks after completing 2-4 cycles of postoperative adjuvant chemotherapy
9. No signs of tumor recurrence before treatment;
10. Hematology, biochemistry, and organ function:

- Hemoglobin ≥ 100 g/L (can be maintained or exceeded this level through transfusion);
- Absolute neutrophil count ≥ 2.0 × 10^9^/L;
- Platelet count ≥ 100 × 10^9^/L;
- Total bilirubin ≤ 1.5 times the upper limit of normal;
- Alanine aminotransferase and aspartate aminotransferase ≤ 2.5 times the upper limit of normal;
- Creatinine ≤ 1.25 times the upper limit of normal; and creatinine clearance rate ≥ 60 mL/min;

1. Women of childbearing age (15-49 years) should have a negative urine pregnancy test within 7 days before starting treatment;
2. Obtain informed consent signed by the patient or their legal representative;
3. Male and female patients of childbearing age agree to use reliable contraceptive methods before entering the trial, during the study, and until 8 weeks after discontinuation of the drug.

### Exclusion criteria

1. Received any other systemic anti-cancer treatment for NSCLC besides 2-4 cycles of adjuvant chemotherapy, including cytotoxic drug treatment, targeted drug treatment (including tyrosine kinase inhibitors or monoclonal antibodies), or experimental treatment;
2. Received local radiotherapy for NSCLC;
3. Physiological imperfection of the upper gastrointestinal tract, or malabsorption syndrome, or inability to tolerate oral medication, or active gastrointestinal ulcers;
4. Clinical objective evidence (pathological or imaging) of tumor recurrence before the start of adjuvant treatment;
5. Patients who have had other cancers besides NSCLC within the five years prior to the start of treatment in this study, excluding cervical carcinoma in situ, cured basal cell carcinoma, bladder epithelial tumors [including Ta and Tis];
6. Known allergy to icotinib or any component of this product;
7. History of interstitial lung disease, drug-induced interstitial lung disease, or any clinically evident active interstitial lung disease;
8. Idiopathic pulmonary fibrosis found on the baseline CT scan;
9. Uncontrolled ocular inflammation or infection, or any condition that may lead to the aforementioned ocular diseases;
10. Any unstable systemic disease, including: active infection, uncontrolled hypertension, unstable angina, angina pectoris that began within the last 3 months, congestive heart failure (≥ New York Heart Association [NYHA] II grade), myocardial infarction (within 6 months before enrollment), severe arrhythmia requiring medication, liver, kidney, or metabolic diseases;
11. Known human immunodeficiency virus (HIV) infection;
12. Patients with mixed small cell lung cancer components;
13. Pregnant or breastfeeding women;
14. History of definite neurological or psychiatric disorders, including epilepsy or dementia;
15. Other conditions deemed unsuitable for enrollment by the investigator.

### Definition of R0 resection:

### Hilar and mediastinal lymph nodes (N1 and N2 lymph nodes) must be excised, marked, and sent for pathology in addition to complete removal of the primary lung lesions.

### At least 3 mediastinal drainage areas (N2 groups) must be sampled and dissected by making the best use of en bloc resection of lymph nodes. Preferably, for the right chest, dissection should include 2R, 3a, 3p, 4R, and 7-9 groups of lymph nodes and surrounding soft tissues; and for the left chest, dissection should include 4L and 5-9 groups of lymph nodes and surrounding soft tissues.

## Study duration

It is planned to enroll a total of 318 patients, and based on an estimated enrollment of about 5 patients per month, the enrollment process is expected to take approximately 64 months; the minimum survival follow-up period is 5 years. The total study period is expected to be about 124 months.

## Withdrawal from study

### Withdrawal criteria

Patients can withdraw from the study treatment and assessment at any stage of the study.

#### Withdrawal time and method

The reasons for withdrawal are as follows:

1. Voluntary withdrawal: Patients can freely withdraw from the trial at any time without affecting future treatment;
2. The investigator and sponsor believe there are any safety reasons (adverse events);
3. The investigator and sponsor believe the patient has poor compliance with the study protocol;
4. Death;
5. Patient lost to follow-up;
6. Other situations deemed appropriate for withdrawal by the investigator.

The investigator considers that the patient must withdraw from the study in the following situations:

1. Receiving other anti-tumor treatments outside of the study protocol during treatment;
2. Severe allergic reactions to the trial drug, such as exfoliative dermatitis or grade 3 to 4 hypersensitivity reactions;
3. Any other serious adverse reactions that the principal investigator or designated researchers believe

require interruption of treatment;

1. Patients with severe poor compliance;
2. The patient's urine β-HCG test result indicates pregnancy. The investigator should report the patient's pregnancy in the form of a clinical trial pregnancy report form;
3. Concurrent other diseases during the study, which, in the judgment of the investigator, will significantly affect the assessment of the patient's clinical situation and require discontinuation of the treatment plan;
4. The occurrence of other malignancies that require treatment;
5. Use of prohibited drugs or other substances that, in the judgment of the investigator, may cause toxicity or bias the study results;
6. Discontinuation of icotinib for more than 2 weeks due to adverse reactions.

#### Withdrawal procedure

For patients who withdraw from the study, the investigator must inquire about the reason for withdrawal and whether any adverse events have occurred. If possible, the investigator should visit and assess the patient who withdraws from the study. The reason and date of withdrawal (the date of the last dose of medication) must be recorded on the Case Report Form (CRF). The patient should return all remaining study medication.

When withdrawing from the trial, if there are new or worsened CTCAE grade 3 or 4 laboratory test values, the patient must undergo further examination, and the results should be recorded in the corresponding section of the CRF until the laboratory test values return to CTCAE grade 1 or 2, unless the test values cannot improve due to the disease itself. For these cases, the investigator must record their opinions in the CRF and medical records.

All existing study-related toxicities and SAEs must be followed up until resolution when the investigator interrupts the study, unless, in the opinion of the investigator, the condition is unlikely to resolve due to the patient's disease itself.

#### Replacement of withdrawn cases

For patients who withdraw from the study early, no replacement will be made.

## Study plan

### Methods for assigning treatment groups

Eligible patients will be centrally randomized to receive icotinib orally for 12 months, icotinib orally for 6 months, or to undergo observation in a 1:1:1 ratio. Patients in the icotinib groups will receive 125 mg orally three times a day after adjuvant chemotherapy for 12 or 6 consecutive months, until disease recurrence or intolerable toxicity occurs. The control group will only undergo observation.

### Withdrawal of icotinib

- Icotinib is well tolerated in clinical monotherapy and generally does not require discontinuation. The incidence of interstitial lung disease (ILD) in Eastern populations treated with gefitinib and erlotinib has been reported to be 2-3% and 1-2%, respectively. ILD was not observed in the ICOGEN clinical study.
- Patients with ILD usually present with acute dyspnea, accompanied by cough, low-grade fever, respiratory discomfort, and arterial blood oxygen desaturation. In the short term, the symptoms can become severe and lead to death. Radiography often reveals pulmonary infiltration or interstitial ground-glass opacity.
- The physician should monitor patients closely for signs of ILD during treatment, and if the patient develops a new acute episode or progressive dyspnea or cough, icotinib therapy should be discontinued and relevant tests should be performed immediately. When ILD is confirmed, medication should be discontinued, and the patient should be treated accordingly.

### Combined therapy and smoking

All drug combinations and treatments (including start/end dates and indications) must be recorded in the patient's original data and in the appropriate section of the CRF.

All patients taking drugs metabolized by CYP3A4 need to be closely monitored for possible adverse effects of these drugs. Smoking can affect the pharmacokinetics of icotinib, and patients' smoking status, including the number and duration of daily smoking, should be recorded during treatment. All patients are advised to quit smoking during treatment.

### Concomitant medication

#### Drugs not allowed to be used

Bevacizumab and any drugs that target VEGF, VEGFR or EGFR (including registered or investigational drugs).

Any anti-cancer drug treatment other than icotinib specified in the study is not permitted, including investigational drugs (such as investigational antibiotics, antiemetics, etc.) and Chinese herbal medicines with anti-tumor characteristics.

#### Permitted drugs

Non-anticancer Chinese herbal therapy or acupuncture, vitamins/micronutrients are allowed to be used without affecting the study endpoint observation, at the discretion of the investigator.

Patients receive palliative and supportive care for pre-existing conditions.

### Compliance

The icotinib dose and date of administration for each patient for each course of treatment should be recorded in the CRF. Reasons for delayed dosing, reduction, or missed dosing are also recorded in the CRF.

Patient compliance with the treatment and regimen includes voluntary compliance with all aspects of the regimen, including compliance with the medication, compliance with all blood sampling required for evaluation of safety, compliance with regular follow-up, etc. Patients who do not take medication on time, or do not cooperate with examinations, or do not return visits on time may be excluded from the study based on the opinion of the lead investigator. Patients who discontinue icotinib due to adverse events that are unmitigated even with optimal symptomatic and supportive care for longer than 2 weeks are withdrawn from the study after discussion with and consent of the principal investigator.

## Study endpoint

The primary efficacy endpoint of the study is DFS according to investigator assessment.

Secondary endpoints include: OS, BMFS, safety and tolerability, and QoL.

Exploratory analysis: potential biomarker exploratory research.

### Efficacy endpoint

DFS is defined as the time between the random date and the first confirmed recurrence of disease or death from any cause, whichever occurs first. DFS rates at 2 and 5 years after randomization are defined as the probability that patients have not relapsed at 2 and 5 years after randomization.

OS is defined as the time between the random day and death from any cause. The 1-year overall survival rate is the probability that a patient is still alive at 1 year from the start of the randomization date. OS rates at 2 and 5 years after randomization are defined as the probability that patients are still alive at 2 and 5 years after randomization.

Patients who are alive and do not have disease recurrence as of the date of analysis have the date of their last imaging assessment as the cut-off time.

### Security specifications

All patients treated with the study drug at least once and those in the observation group will be included in the safety analysis. The patient's physical exam results, vital signs, adverse events, and laboratory test outliers are summarized. Adverse events should be reported and graded according to the NCI Common Adverse Events Terminology Standard (CTCAE) version 4.0.

## Study process (See Table 1)

### Screening period

**Screening period 1 - within 28 days before starting the study drug**

**·**Sign informed consent prior to performing any research-related operations;

**·**Demographic information, complete medical, surgical, and smoking history;

**·**Recording/confirming the TNM stage of the primary tumor at diagnosis (7th edition TNM staging);

**·**Tumor evaluation during screening: chest and upper abdominal contrast CT, brain contrast MRI/CT, and bone scan;

**·**All tests can be replaced by PET/CT;

**·**Tumor tissue sample acquisition and EGFR detection. EGFR mutations (exon 19 deletion or L858R mutation in exon 21) will be assessed by the central laboratory using the amplification refractory mutation system (ARMS).

**Screening period 2 - within 7 days before starting treatment with the study drug**

- Confirm that subjects meet the inclusion/exclusion criteria specified in the study protocol;
- Physical examination: heart rate, blood pressure, respiratory rate, body temperature, height, weight and ECOG performance status;
- Record all concomitant diseases and drug combinations and their indications;
- Blood routine tests: hemoglobin, hematocrit, platelet count, white blood cell count, neutrophils, etc.;
- Blood biochemical test: blood glucose, calcium, phosphorus, sodium, potassium, chlorine, creatinine, blood urea nitrogen (BUN), total protein, albumin, alanine aminotransferase (ALT), aspartate aminotransferase (AST), alkaline phosphatase, total bilirubin, etc.;
- Baseline Electrocardiogram (ECG) examination, examined and signed by the investigator;
- Urine pregnancy tests are recommended to be performed on all women of childbearing age. Postmenopausal women who have been through menopause for at least 2 years and women who have undergone sterilization are not required to undergo a urine pregnancy test;
- Urine routine test: pH value, urine sugar, urine protein, ketone body, occult blood, urine bilirubin, red blood cells, white blood cells, etc.;
- HIV antibody test;
- Lung function test;
- Tumor marker examination;
- Biomarker sample collection (optional): 10ml whole blood for biomarker analysis;
- Adverse events: Signs and symptoms present prior to enrollment (prior to signing of the informed consent) and persisting at enrollment should be documented by medical history. Any signs and symptoms that occur or worsen after enrollment (even before initiation of medication) should be recorded as adverse events according to NCI-CTCAE version 4.0;
- Study drug delivery.

### Treatment period

**Treatment period visits (every 30 days ±7 days)**

- Perform a physical examination (heart rate, blood pressure, respiratory rate, body temperature, weight and ECOG performance status);
- QoL assessment: FACT-L and LCSS questionnaires;
- Laboratory examination (hematology/blood biochemistry/urine routine); ECG;
- Biomarker sample collection (optional): 10 ml of whole blood is collected and stored for biomarker testing before treatment;
- Tumor evaluation: chest and upper abdominal contrast CT should be included. Patients are scheduled for visits of assessment once a month during the treatment period, every 3 months for the first 2 years after randomization, every 6 months until 5 years, and every 12 months thereafter until disease recurrence or death. If new lesions are suspected at any site, CT should be performed until the disease recurs. Brain MRI and bone scans are scheduled every 12 months or performed as indicated based on symptoms. All imaging tests can be replaced by PET/CT;
- Record adverse events and drug combinations since the last visit;
- Schedule next visit.

**End-of-treatment/end-of-study visit**

If a subject discontinues study medication for any reason (other than death or loss of follow-up), evaluation should be performed at the time of discontinuation of medication, including:

1. Physical examination and vital signs examination, ECOG performance status;

2. Laboratory tests: Complete blood count (CBC), biochemical test, urine test;

3. Electrocardiogram;

4. Record of adverse events: If adverse events persist at the end of treatment, or new adverse events are judged by the investigator to be related to the investigational drug, information about adverse events should be collected until 30 days after the end of treatment (can be collected by telephone interview). Unless the investigator believes that the occurrence of an adverse event is caused by the patient's other disease, follow-up should continue until the adverse event resolves or stabilizes;

5. Records of drug combinations;

6. Biomarker sample collection (optional): 10ml whole blood for biomarker analysis.

### Survival follow-up

If the patient relapses, telephone survival follow-up is conducted every 3 months after recurrence until 5 years after surgery or death. The following information should be obtained during each follow-up visit:

- Whether the patient survives.
- If deceased, record the date and cause of death in detail.
- Disease status, with a detailed record of the date of recurrence if the patient relapses after surgery (for patients who have not relapsed at the last follow-up).
- Keep detailed records of subsequent anti-cancer treatments.

Note: Patients who have experienced tumor recurrence will be judged by the investigator and followed up with local medical practice.

### Temporary visit

Temporary visits should be made according to clinical needs. Relevant clinically significant laboratory abnormalities and adverse events should be recorded in the CRF. If multiple laboratory tests are performed on the same day, record the last set of test values in the CRF. However, outliers in all repeated laboratory tests should be recorded in the CRF.

## Quality of data

To adhere to the guidelines of Good Clinical Practice (GCP), monitors will visit each site regularly to ensure compliance with the study protocol, GCP, and relevant laws. Visits will include on-site inspections of the completeness and clarity of CRFs, cross-checking of CRFs with source documents, and resolution of data queries.

## Archiving

The information entered into the CRFs must be consistent with the original documents. Study documents and all original materials should be retained for 15 years after the end of the trial until a written notice of destruction is received from the sponsor.

# INVESTIGATIONAL DRUG

## Icotinib

### Clinical trial drug name, physicochemical properties, appearance, specifications, composition, usage, storage

**Drug name**

Trade name: Conmana®

Generic name: Icotinib Hydrochloride Tablets

Chemical name: 4-[(3-ethynylphenyl) amino] quinazoline[6,7-b]-12-crown-4 hydrochloride

Its structural formula is:

Molecular formula: C_22_H_21_N_3_O_4_·HCl

Molecular weight: 427.88

**Physicochemical properties of the drug**

Characteristics of physicochemical properties: Icotinib hydrochloride is a white to off-white crystalline powder; odorless and non-hygroscopic. It is soluble in dimethyl sulfoxide, slightly soluble in acetonitrile-water (1:1), methanol, or chloroform, very slightly soluble in ethanol, and practically insoluble in water and acetonitrile. The melting point is 225-228℃. The absorption coefficient （E） at a wavelength of 340 nm is 500-520.

**Drug appearance:** This product is a brownish-red film-coated tablet, which appears white after the coating is removed.

**Drug specification:** 125mg.

**Drug composition:** The main ingredient of this product is icotinib hydrochloride.

**Drug usage:** Oral administration, one tablet each time, three times a day.

**Drug storage:** Store in light-shielding and sealed conditions.

### Drug packaging and labeling

The investigational drug is packaged in an aluminum-plastic plate and placed in a pre-labeled medication box for administration to the subjects. Each medication box label contains the protocol number, patient code, drug batch number, dosing instructions, and the name of the manufacturer, indicating: "Not for sale, for clinical research only."

### Drug distribution and inventory

The investigational drug should be dispensed by the research personnel.

After an appropriate interval, or until the end of the study, all unused drugs as well as empty boxes and drug plates must be returned to the sponsor. At the end of the study, the monitor should check all unused items.

An inventory list must be kept for the monitor to inspect.

# ETHICAL AND LEGAL ASPECTS

## Independent ethics committee (IEC)

According to GCP, China's laws and regulations, and the requirements of relevant organizations, all centers participating in the study should obtain the approval documents from the corresponding ethics committee before the start of the study. If necessary, amendments or re-reviews must be obtained from the ethics committee and forwarded to the investigators.

## Ethical guidelines for this study

The procedures related to operations, evaluations, and document preparations involved in this study protocol are designed to ensure that researchers follow the clinical practice guidelines and the guiding principles detailed in the Declaration of Helsinki. The implementation of this study will also adhere to China's corresponding "Good Clinical Practice".

Without the written consent of the ethics committee and the sponsor, researchers cannot modify the study protocol. However, in urgent cases to eliminate risk factors for patients, researchers may deviate from or change the study protocol before obtaining consent/support from the ethics committee/institutional review board/sponsor. Any deviations or changes made and their reasons should be submitted to the ethics committee/institutional review board/sponsor as soon as possible, and if appropriate, suggestions for protocol modifications should also be submitted. Researchers must fully explain and justify all deviations or changes from the study protocol.

## Patient information and informed consent

Patients should be provided with the main information about the study and the informed consent form. Before the start of the study, researchers must provide patients with the informed consent form and all other written information that has been approved in writing by the ethics committee. The ethics committee approval letter and the approved patient information/informed consent form must be archived together in the study files.

Before implementing any specific steps related to this study, the signed informed consent form must be obtained from the patient.

## Confidentiality

All records related to patient identity shall be kept confidential and, within the limits permitted by relevant laws and/or regulations, these data will not be disclosed to the public. Only personnel related to the trial, such as investigators and research nurses, may know the identity of the patients.

The patient's name will not appear in the CRF. The CRF will only record the patient's number and the initials of their name. If the patient's name appears in any other documents (such as pathology reports), it must be erased when copies of the documents are made. Computer-stored study reports must comply with local data protection laws. When the results of the study are published, the identity of the patients will also be kept confidential.

The investigator will maintain a list to identify patient records.

## Conditions for amending the study protocol

Amendments to the ongoing trial protocol can only be made after consultation between the sponsor and the principal investigator. The sponsor prepares the materials for the revised protocol, which should be reviewed in advance by the principal investigator, biostatistician, and other relevant personnel. Unless it is an emergency adjustment to eliminate harm to trial patients, or it is just a logistical and administrative adjustment related to the trial, such as changes in monitors or telephone numbers, all protocol amendments must be submitted to the corresponding ethics committee and obtain its permission. If necessary, they must also be submitted to the drug regulatory authorities, and the researchers can only implement the adjustments after permission from the above units.

## Conditions for terminating the study

The sponsor and the principal investigator reserve the right to terminate the study at any time. When terminating the study, both the sponsor and the principal investigator will ensure that the interests of the patients are fully considered.

## Preservation of study documents, case report forms, and records

### Preservation of investigator documents

To ensure that the implementation of the study is fully recorded and that the study data can be subsequently verified, the investigator must retain comprehensive and accurate records during the conduct of the study. These documents should be divided into two different categories: (1) investigator's study documents, and (2) patient clinical source records.

The investigator's study documents include the trial protocol and revisions, approval documents from independent ethics committees and governments, informed consent form samples, drug-related records, personnel resumes, and other relevant documents/communications, etc.

Clinical source documents (typically predefined before the study begins and recorded outside of the CRFs, containing critical efficacy/safety parameters), usually including inpatient/outpatient records, physicians’ and nurses’ notes, appointment schedules, original laboratory reports, electrocardiograms (ECGs), imaging reports, pathology reports, and specialized assessment reports, signed informed consent forms, consulting letters, patient screening and enrollment forms. At least 15 years after the study is completed or terminated, researchers must retain both types of documentation. After this period, these documents may be destroyed in accordance with local procedures. If investigators intend to transfer these study records to another organization or ship them to another location, they must notify the sponsor in advance.

If the investigator cannot guarantee the archiving requirements for any or all documents at the research site, the investigator and the sponsor should make special arrangements to seal and store these materials off-site, so that if there is a regulatory audit, the sealed documents can be returned to the investigator. If the patient needs the original records for subsequent treatment, then the corresponding photocopies should be preserved outside the research center.

### Original records and background data information

Investigators should provide any background data information required by the sponsor from the study documents and clinical record data as needed, especially when there is a suspicion of errors occurring in data transcription, which is even more important. It should also be necessary to be able to access the complete study records in the event of special problems and/or government inquiries or requests for audit inspections, while at the same time ensuring the protection of patient privacy rights.

### Audit and inspection

Investigators should be aware that upon receiving formal notice, they should prepare the original records related to the study and provide them to the qualified personnel or their designated individuals, or to the inspectors from the health department. The inspection of data in the CRFs must be directly checked against the original records.

### Case report forms

For each enrolled patient, the Case Report Forms (CRF) must be completed and signed by the principal investigator or a representative authorized by the principal investigator or his/her delegates. This also applies to the CRF records of patients who failed to complete the trial (even during the screening period, if the CRF has been filled out). If a patient withdraws from the treatment study, the reason for withdrawal must be recorded on the CRF. If a patient withdraws from the study due to a treatment-related adverse event, a comprehensive effort should be made to clearly document the outcome. Investigators should ensure the accuracy, completeness, legibility, and timeliness of the data reported in the CRFs and all required reports to the sponsor.

# MONITORING OF THE STUDY

If patient confidentiality meets local requirements, the responsible monitor (or designee) will regularly contact and visit the investigator and be allowed to inspect various trial records (CRFs and other relevant data) as required.

Throughout the entire study period, the monitor is responsible for regularly checking the CRFs, verifying compliance with the study protocol, and checking the completeness, consistency, and accuracy of the entered data. The monitor should have access to laboratory test reports and other patient records to verify the entries on the CRFs. Investigators (or their designated personnel) agree to cooperate with the monitor to ensure that any issues identified during these monitoring visits are resolved.

# PUBLICATION OF RESEARCH DATA AND PROTECTION OF TRADE SECRETS

The results of the study may be published or presented at scientific conferences. If this is foreseeable, the investigator should commit to submitting all manuscripts or abstracts to the sponsor before submission, which allows the sponsor to protect proprietary information. At the same time, it helps the sponsor to provide corresponding suggestions for the investigator based on information from other studies that the investigator cannot obtain. In accordance with publishing standards and ethical guidelines, support the publication of overall research data from multicenter studies rather than data from individual centers. In such cases, a coordinating investigator will be appointed following mutual consent.

# STATISTICAL AND ANALYSIS PLAN

## Primary and secondary variables

### Primary variable

The primary efficacy endpoint is DFS according to investigator assessment.

DFS is defined as the time from randomization to the first confirmed recurrence of disease or death from any cause (whichever occurs first). Patients who have no disease recurrence and no death at the end of the study (or data cutoff), or who are lost to follow-up after randomization, will be censored at the date of the last tumor assessment confirming no recurrence after randomization.

### Secondary variables

Secondary endpoints include: OS, BMFS, safety, tolerability, and QoL.

OS is defined as the time from randomization to death due to any cause. Patients who are still alive at the time of analysis will be censored at the date of their last contact.

BMFS is defined as the time from randomization to brain metastasis or death, whichever comes first. The expected survival curves for the treatment groups will be derived through the Kaplan-Meier method.

QoL will be assessed with the Functional Assessment of Cancer Therapy – Lung (FACT-L) questionnaire and Lung Cancer Symptom Scale (LCSS).

## Statistical analysis

### Baseline and demographic characteristics

Baseline data include: demographic characteristics, tumor staging, medical history, surgical procedures, concomitant medications, vital signs, etc. Dealing with continuous data will involve using descriptive statistics such as mean, standard deviation, median, minimum, and maximum; for categorical variables, frequencies and percentages will be used. More details will be stated in the separate Statistical Analysis Plan.

### Primary efficacy

The primary efficacy variable of this study is the DFS according to investigator assessment.

### Secondary efficacy

The analysis of secondary efficacy variables includes the following:

- OS.
- BMFS.
- Safety analysis:. Adverse events and their most severe reaction levels will be summarized according to the standards of NCI-CTCAE version 4.0, and adverse events will also be summarized based on the severity of the events and their relationship with the study drug. Descriptive summaries of laboratory test values will mainly focus on abnormal values. Laboratory abnormalities will also be summarized according to the most severe level in NCI CTCAE version 4.0.
- QoL: QoL will be assessed with the FACT-L questionnaire and LCSS.

### Exploratory analysis

- This study plans to conduct exploratory analyses of relevant biomarkers.

## Type of analysis

Intention-to-treat population

All statistical analyses will be conducted in the intention-to-treat (ITT) population, which is defined as all patients who have been enrolled.

Per-protocol population

The per-protocol (PP) population will consist of patients who complete planned treatments, including patients who complete 12 or 6 months of icotinib, and patients in the observation group.

Safety analysis population

All patients who receive at least 1 dose of the study treatment in the 12-month or 6-month icotinib group and all those in the observation group will be evaluated for safety and tolerability.

## Interim analysis

This study has a planned interim analysis. Interim analysis will be conducted when the DFS events reach 50% (99/198) for final analysis. More details about the statistical methods will be stated in the separate Statistical Analysis Plan.

## Safety analysis

For all patients in the safety population, the following safety parameters will be analyzed and presented based on the treatment received:

- Adverse Events;
- Serious Adverse Events;
- All adverse events ≥ grade 3;
- All adverse events leading to treatment discontinuation;
- All adverse events leading to dose reduction;
- Fatal adverse events.

Adverse events and their most severe reaction levels will be summarized according to the NCI-CTCAE version 4.0 criteria. Adverse events will also be summarized based on the severity of the events and their relationship with the study drug. Descriptive summaries of laboratory values will focus on abnormal values. More details will be stated in the separate Statistical Analysis Plan.

# PRINCIPLE OF SAFETY

## Precautions/Warnings

**Icotinib**

The common adverse drug reactions of icotinib include: rash (39.5%), diarrhea (18.5%), and elevated transaminases (8.0%). The majority are grade I-II, generally occurring within 1-3 weeks after medication, and are usually reversible without special treatment, disappearing on their own.

**The warnings and precautions for icotinib are as follows:**

1. According to literature reports, the incidence of interstitial lung disease (ILD) in Eastern populations treated with gefitinib and erlotinib is 2-3% and 1-2%, respectively. No cases of interstitial lung disease were observed in the ICOGEN clinical study. Patients with interstitial lung disease typically experience acute dyspnea, accompanied by cough, low-grade fever, respiratory discomfort, and arterial oxygen desaturation. These symptoms can develop severely in a short period and lead to the patient’s death. Radiological examinations often show pulmonary infiltration or interstitial ground-glass opacity.

During treatment, the physician should closely monitor patients for signs of ILD. If a patient experiences a new acute onset or progressive worsening of dyspnea or/and cough, the treatment with this product should be interrupted, and relevant tests should be carried out immediately. When ILD is confirmed, medication should be discontinued, and the patient should receive corresponding treatment.

According to literature reports, high-risk factors for developing ILD include: smoking, poor performance status (PS≥2), ≤50% normal lung tissue coverage on CT scans, short time since diagnosis of non-small-cell lung cancer (<6 months), pre-existing interstitial pneumonia, older age (≥55 years), and accompanying heart disease. Patients with the above high-risk factors should be cautious when using this product.

1. A transient mild increase in hepatic transaminases has been observed in a small number of patients, and these patients should use this product with caution. Patients with moderate or higher increases in transaminases need to interrupt medication, monitor transaminases until the increase is alleviated or disappears before resuming medication.

3. Seek immediate medical attention if the following conditions worsen: a new acute onset or progressive worsening of dyspnea, cough; severe or persistent diarrhea, nausea, vomiting, or anorexia.

4. Impact on the ability to drive and operate machinery: During treatment with this product, symptoms of fatigue may occur, and patients with these symptoms should be reminded to be cautious when driving or operating machinery.

Icotinib may produce clinically significant drug-drug interactions (see icotinib instructions for details).

## Adverse events

### Definition of adverse events

An adverse event (AE) is any unfavorable medical occurrence in a patient or patient treated with a study drug. AEs do not necessarily have a causal relationship with the drug. Therefore, AEs can be any unfavorable and unexpected signs (including abnormal laboratory findings), symptoms, or diseases temporally associated with the use of the investigational drug, regardless of whether the event is considered related to the drug. Conditions where pre-existing symptoms worsen during the study period can also be reported as AEs.

AEs occurring in human subjects (whether drug-related or not) include the following aspects:

- AEs occurring during the process of drug use;
- AEs caused by drug overdose (intentional or unintentional);
- AEs caused by drug abuse;
- AEs caused by discontinuation of medication;
- AEs that may be purely due to the patient's participation in the study, even if unrelated to the study drug, must be reported as AEs.

The absence of or failure to achieve the expected clinical pharmacological action is not considered an AE.

### Adverse event grading

All AEs will be graded according to the National Cancer Institute (NCI) Common Terminology Criteria for Adverse Events version 4.0 (CTCAE 4.0) through a 5-point scale (Grades 1-5), and detailed reporting will be included in the CRF.

### Monitoring of adverse events

Patients must be closely monitored for AEs. This monitoring includes clinical laboratory tests. AEs should be evaluated based on their severity, seriousness, and relationship with the investigational drug.

The investigator is responsible for assessing the relationship between all AEs and the study drug. However, the principal investigator may delegate this judgment to other investigators participating in the study, but they are still responsible for it.

### Relationship between adverse events and the study drug

The evaluation of the relationship between an AE and the study drug is a comprehensive clinical judgment made based on all the information obtained at the time of completing the CRF.

Situations assessed as "unrelated" may include: a clear alternative explanation, such as traumatic bleeding at the surgical site; unreasonable, such as a patient being hit by a car, but there are no signs that the event was caused by drug-induced disorientation; or cancer occurring just a few days after the start of medication.

An assessment of "related" indicates that there is a reasonable reason to suggest that the AE may be related to the study medication.

Factors to consider when evaluating the relationship between AEs and the study drug include:

- Appearance shortly after drug use: The AE should occur shortly after administration of the drug. The clinical evaluation of the event should consider the duration between medication and the occurrence of the event.
- Disappearance of the event after stopping medication, and recurrence of the event after re-administration: The clinical course of the suspected event should fully consider the patient's response after stopping medication or after the patient takes the medication again.
- Underlying diseases, concurrent diseases, and intercurrent diseases.
- Concomitant medications or treatments: It is necessary to check other drugs taken by the patient or other treatments received to determine if one of them may have caused the AE.
- Known reaction patterns of certain classes of drugs: Clinical/preclinical.
- Pharmacology and pharmacokinetics of the test drug: The pharmacokinetic characteristics (absorption, distribution, metabolism, and excretion) of the test drug should be considered in conjunction with the individual pharmacodynamic response of each patient.

### Definitions of serious adverse events

A serious adverse event (SAE) refers to any untoward medical occurrence that occurs at any dose that meets one of the following conditions:

- Results in death;
- Life-threatening events;
- Results in hospitalization or prolongation of existing hospitalization;
- Results in persistent or significant disability/incapacity;
- Important Medical Events.

Life-threatening: The term "life-threatening" is defined as "serious", indicating that the patient was at risk of death at the time of the AE. It does not refer to those AEs that might have caused death if the situation had been more severe.

Hospitalization: Any AE that results in hospitalization or prolongation of existing hospitalization is considered serious, unless it meets one of the following exceptions: observation in hospital for not more than 12 hours; or admission was pre-planned; or admission is unrelated to the AE.

***Note:*** Any invasive treatment during hospitalization may meet the criteria for an "Important Medical Event" and may need to be reported as an SAE according to clinical judgment. Moreover, if local regulatory authorities specifically require a stricter definition, local regulations shall prevail.

Disability: Means that a person's ability to perform daily activities is severely impaired.

According to the standard definition, the term sudden death can only be used when the cause of death is cardiac. The terms death and sudden death are clearly different and should not be used interchangeably.

Any clinical AE or abnormal laboratory test value that reaches a serious level (according to the above definition) occurring during the study must be reported to the sponsor, drug regulatory authorities, and ethics committee, within one working day after the investigator becomes aware of the event, regardless of what treatment the patient receives.

SAEs related to study treatment must be collected and reported, no matter how long after the last dose, even if the trial has ended.

SAEs unrelated to study treatment must be collected and reported during the study period and within 28 days after the last dose of the trial.

### Malignant tumor progression

**In this study, disease progression and death caused by disease progression are not reported as SAEs**

If the progression of a potentially malignant tumor is clearly consistent with the suspicious progression of the potential cancer defined by the RECIST 1.1 criteria or other criteria specified in the study protocol, it is not reported as an AE. Mere hospitalization due to the progression of a potential malignant tumor is not reported as an SAE. If it cannot be determined whether these symptoms are due to the progression of a potential malignant tumor or if they do not conform to the expected progression pattern of the study disease, the clinical symptoms of progression can be reported as an AE.

Some patients' symptoms may worsen. In such cases, there is a clear progression of the patient's clinical symptoms, but the tumor measurement results do not support disease progression. Or the disease progression is so obvious that researchers may choose not to conduct further disease assessments. In such cases, clinical progression will be determined based on the deterioration of symptoms. These situations of determining clinical progression should be rare exceptions, as every effort should be made to determine the objective progression of potential malignant tumors.

If it is uncertain whether an AE is due to the study disease, it should be reported as an AE or SAE.

**Important Medical Events:** Any AE that may harm the patient and may require intervention to prevent a more serious situation can be considered a SAE. Please refer to the 'World Health Organization Adverse Reaction Terminology - Main Term List' to determine important Medical Events. These terms refer to serious disease states or descriptions of serious disease states. Such events are reported as SAEs because they may be related to serious disease states, and reporting them as SAEs ensures special attention compared to other reporting methods and promotes necessary actions.

### Unexpected adverse events

Unexpected AEs refer to any drug adverse reaction whose characteristics or severity are inconsistent with the investigator's brochure. Serious unexpected adverse reactions refer to unexpected SAEs related to the investigational drug.

### Reporting of adverse events

AEs that occur after the patient signs the informed consent form until 28 days after the last dose of medication should be recorded in detail.

The documentation must be supported by original data. Laboratory abnormalities that are considered clinically relevant (for example, those leading to the patient's early withdrawal from the study, requiring treatment, or causing significant clinical manifestations, or those deemed clinically relevant by the investigator) should be recorded as AEs. Each event should be described in detail, including start and end dates, severity, relationship with the study drug, measures taken, and the outcome of the event.

SAEs that meet the definition occurring from the signing of the informed consent form until 28 days after the last dose, including laboratory abnormalities that meet the definition of SAEs, must be reported immediately (within 24 hours after the investigator becomes aware of them) to the designated person in the study documents. The SAE report form must also be completed and submitted to the designated person within 24 hours after the investigator becomes aware of an SAE. SAEs related to study treatment, regardless of how long after the last dose, even if the trial has ended, must be collected and reported. Disease progression in this study is not reported as an SAE (see Section 9.2.6).

Each SAE should be followed up until resolution or stability, and updated reports should be submitted to the designated personnel. Isolated Grade 4 laboratory abnormalities (according to CTC-AE version 4.0 standards) should not be reported as serious adverse reactions unless the investigator believes that the abnormalities meet the criteria for SAE (see Section 9.2.5). Grade 5 laboratory abnormalities according to CTC-AE version 4.0 standards that appear during the baseline period and are manifestations of the disease should not be reported as SAEs, especially when patients with these abnormalities are present and still allowed for enrollment by the protocol or not excluded from enrollment. If there is doubt about whether such an abnormality should be reported as an SAE, the investigator may consult the study monitor. Grade 4 laboratory abnormalities according to CTC-AE should be recorded on the 'Laboratory Data' page and regularly reviewed by the medical monitor. If it is uncertain whether an AE is due to the study disease, it should be reported as an AE or SAE.

According to local laws and regulations, SAEs must be reported to the ethics committee and the drug regulatory authorities.

### Treatment and follow-up of adverse events

The outcome of each AE must be recorded on the CRF. All AEs will be followed up according to the following guidelines:

**Treatment-related AEs**

Continue follow-up until one of the following outcomes occurs:

- Resolution or improvement to the baseline level.
- Causality is reassessed as unrelated.
- Death.
- Initiation of a new anticancer regimen.
- The investigator confirms that further improvement is not expected.
- No further clinical or safety data collection will be conducted or the final database is closed.

**Unrelated severe or life-threatening AEs**

Continue follow-up until one of the following outcomes occurs:

- Resolution or improvement to the baseline level.
- Severity improves to Grade 2.
- Death.
- Initiation of a new anticancer regimen.
- The investigator confirms that further improvement is not expected.
- No further clinical or safety data collection or the final database is closed.

**Unrelated grade 1 or 2 AEs:**

Continue follow-up until one of the following outcomes occurs:

- Resolution or improvement to the baseline level.
- Initiation of new anticancer treatment.
- The investigator confirms that further improvement is not expected.
- No further collection of clinical or safety data, or the final database is closed.

### Laboratory test value abnormalities

Record laboratory test results in the CRF. For any laboratory result abnormality that meets SAE criteria, in addition to recording it as an AE in the CRF, it should also be reported as an SAE.

In the AE page of the CRF, any clinically significant abnormal laboratory test results caused by treatment should be recorded in the form of a single diagnostic result, that is, these results meet one or more of the following conditions:

- Clinical complications have occurred.
- It leads to changes in the study medication (such as dose changes, interruption of administration, or permanent discontinuation).
- It requires changes in the concurrent treatment plan (such as increases, interruptions, discontinuations, or other changes to concurrent drugs, treatments, or medication measures).

The above apply to any protocol and non-protocol specified laboratory safety and efficacy results obtained from tests conducted after the first dose of study drug, which exceed the laboratory reference range and meet clinical significance criteria.

The above do not apply to any laboratory test result abnormalities that exceed the laboratory reference range but do not meet clinical significance; types of AEs explicitly excluded by the protocol; or results arising from AEs that have been reported or are being reported.

If a medically significant, unexplained laboratory abnormality occurs, the test should be repeated and followed up until it returns to the normal range and/or an appropriate explanation for the abnormality is found. If a clear explanation has been obtained, it should be recorded on the CRF.

## Pregnancy

If a female patient becomes pregnant during the study, she must stop using the study medication as instructed and immediately notify the investigator. The investigator must report the pregnancy outcome to the sponsor and drug regulatory authorities, the ethics committee, within 24 hours through the clinical trial pregnancy report form. The investigator should provide medical counseling to the patient and discuss the risks of continuing pregnancy and the potential impact on the fetus. The patient should be monitored until the end of pregnancy, and if the baby is born alive, the baby should also be followed up. Pregnancies that occur within 90 days after the completion of study drug administration should also be reported to the investigator.

If the spouse of a male patient participating in the study becomes pregnant or becomes pregnant within 90 days after the end of the study medication, it is necessary to obtain the informed consent signed by the pregnant spouse through the Pregnant Spouse Data Release Form as much as possible, and follow up and report the pregnancy outcome to the investigator and sponsor. The spouse should be consulted to discuss the risks of continuing pregnancy and the possible adverse reactions to the fetus. The patient should continue to be monitored until the end of pregnancy. If the baby is born alive, the baby should also be followed up.

# INDEPENDENT DATA MONITORING COMMITTEE (IDMC)

The Independent Data Monitoring Committee (IDMC) will meet every year from the trial begins.

The timing and frequency of the IDMC meeting may be changed if the IDMC considers it necessary. The IDMC will make recommendations to continue, amend, or stop the study based on SAEs, adverse events, and other safety data. The IDMC will conduct an interim analysis. If the interim analysis meets the criteria of early termination of the trial, the sponsor and the principal investigator may accept IDMC's recommendation to terminate the study.

The review by the IDMC does not determine the eligibility of patient enrollment, nor does it decide on the patient's treatment. Investigators make all treatment decisions based on local assessment results. The main analyses of OS and DFS are based on investigator assessment results.

# REFERENCES

[1] Parkin DM, Bray F, Ferlay J, et al. Global cancer statistics, 2002. CA Cancer J Clin. 2005; 55:74-108.

[2] Govindan R, Page N, Morgensztern D, et al. Changing epidemiology of small-cell lung cancer in the United States over the last 30 years: Analysis of the surveillance, epidemiologic, and end results database. J Clin Oncol. 2006;24:4539-44.

[3] Goldstraw P, Crowley J, Chansky K, et al. International association for the study of lung cancer international staging committee; participating institutions. The IASLC lung cancer staging project: proposals for the revision of the TNM stage groupings in the forthcoming (seventh) edition of the TNM Classification of malignant tumours. J Thorac Oncol 2007;2:706-14.

[4] Groome PA, Bolejack V, Crowley JJ, et al. IASLC International Staging Committee; Cancer Research And Biostatistics; Observers To The Committee; Participating Institutions. The IASLC lung cancer staging project: validation of the proposals for revision of the T, N, and M descriptors and consequent stage groupings in the forthcoming (seventh) edition of the TNM classification of malignant tumours. J Thorac Oncol 2007;2:694-705.

[5] Goldstraw P, Crowley J, Chansky K, et al. The IASLC Lung Cancer Staging Project: proposals for the revision of the TNM stage groupings in the forthcoming (seventh) edition of the TNM classification of malignant tumours. J Thorac Oncol 2007;2:706-714.

[6] Girard N, Mornex F. Radiotherapy for locally advanced non-small cell lung cancer. Eur J Cancer 2009;45S1:113-25.

[7] Non-Small Cell Lung Cancer Collaborative Group: Chemotherapy in non-small cell lung cancer: A meta-analysis using updated data on individual patients from 52 randomized clinical trials. BMJ 1995;311:899-909.

[8] The International Adjuvant Lung Cancer Trial Collaborative Group: Cisplatin-based adjuvant chemotherapy in patients with completely resected non-small cell lung cancer. N Engl J Med 2004;350:351-60.

[9] Winton T, Livingston R, Johnson D, et al: Vinorelbine plus cisplatin vs. observation in resected non-small-cell lung cancer. N Engl J Med 2005;352:2589-97.

[10] Douillard JY, Rosell R, De Lena M, et al: Adjuvant vinorelbine plus cisplatin versus observation in patients with completely resected stage IB-IIIA non-small cell lung cancer (Adjuvant Navelbine International Trialist Association [ANITA]): A randomised controlled trial. Lancet Oncol 2006;7:719-27.

[11] Scagliotti GV, Roldano F, Torri V, et al: Randomized study of adjuvant chemotherapy for completely resected stage I, II, or IIIA non-small cell lung cancer. J Natl Cancer Inst 2003;95:1453-1461.

[12] Waller D, Peake RJ, Stephens RJ, et al: Chemotherapy for patients with non-small cell lung cancer: The surgical setting of the Big Lung Trial. Eur J Cardiothorac Surg 2004;26:173-82.

[13] Pignon JP, Tribodet H, Scagliotti GV, et al. Lung Adjuvant Cisplatin Evaluation: a pooled analysis by the LACE collaborative group. J Clin Oncol, 2008, 26(21): 3552-59.

[14] Strauss GM, Herndon JE 2nd, Maddaus MA, et al. Adjuvant paclitaxel plus carboplatin compared with observation in stage IB non-small-cell lung cancer: CALGB 9633 with the Cancer and Leukemia Group B, Radiation Therapy Oncology Group, and North Central Cancer Treatment Group Study Groups. J Clin Oncol. 2008;26:5043-51.

[15] Mok TS, Wu YL, Thongprasert S, et al. Gefitinib or carboplatin-paclitaxel in pulmonary adenocarcinoma. N Engl J Med. 2009; 361: 947-57.

[16] Zhou C, Wu YL, Chen G, et al. Erlotinib versus chemotherapy as first-line treatment for patients with advanced EGFR mutation-positive non-small-cell lung cancer (OPTIMAL, CTONG-0802): a multicentre, open-label, randomised, phase 3 study. Lancet Oncol. 2011; 12: 735-42.

[17] Rosell R, Carcereny E, Gervais R, et al. Erlotinib versus standard chemotherapy as first-line treatment for European patients with advanced EGFR mutation-positive non-small-cell lung cancer (EURTAC): a multicentre, open-label, randomised phase 3 trial. Lancet Oncol. 2012; 13: 239-46.

[18] Goss GD, Lorimer I, Tsao MS, et al. A phase III randomized, double-blind, placebo-controlled trial of the epidermal growth factor receptor inhibitor gefitinib in completely resected stage IB-IIIA non-small cell lung cancer (NSCLC): NCIC CTG BR.19. J Clin Oncol. 2010;28(20s Suppl):lba7005.

[19] Richardson F, Richardson K, Sennello G. et al. Biomarker analysis from completely resected NSCLC patients enrolled in an adjuvant erlotinib clinical trial (RADIANT). J Clin Oncol. 2009;27(20s Suppl):abstr 7520.

[20] Janjigian YY, Park BJ, Zakowski MF, et al. Impact on disease-free survival of adjuvant erlotinib or gefitinib in patients with resected lung adenocarcinomas that harbor EGFR mutations. J Thorac Oncol. 2011;6:569-75.

[21] Neal JW, Pennell NA, Govindan R, et al. The SELECT study: A multicenter phase II trial of adjuvant erlotinib in resected epidermal growth factor receptor (EGFR) mutation-positive non-small cell lung cancer (NSCLC). J Clin Oncol 30, 2012 (suppl; abstr 7010).

[22] Wang S, Ou W, Li N, et al. Pemetrexed-carboplatin adjuvant chemotherapy with or without gefitinib in resected stage IIIA-N2 non-small cell lung cancer harbouring EGFR mutations: a randomized, phase II study. J Clin Oncol 31, 2013 (suppl; abstr 7519)

[23] Zhao Q, Shentu J, Xu N, et al. Phase I study of icotinib hydrochloride (BPI-2009H), an oral EGFR tyrosine kinase inhibitor, in patients with advanced NSCLC and other solid tumors. Lung Cancer. 2011. 73(2): 195-202.

[24] Sun Y, Shi Y, Zhang L, et al. A randomized, double-blind phase III study of icotinib versus gefitinib in patients with advanced non-small cell lung cancer (NSCLC) previously treated with chemotherapy (ICOGEN). J Clin Oncol 29, 2011. (suppl; abstr 7522).

# Investigator's Statement

I have read this protocol, and the study will be conducted in accordance with the clinical, ethical, and scientific principles outlined in the Helsinki Declaration and China's GCP. I agree to carry out this clinical study in accordance with the design and provisions of this protocol.

I will be responsible for making medical decisions related to the clinical trial, ensuring that subjects receive timely treatment in the event of an AE during the trial. I am aware of the procedures and requirements for correctly reporting SAEs, and I will record and report these events as required.

I guarantee that the data will be accurately, completely, timely, and legally entered into the CRF. I will accept monitoring or auditing by monitors or auditors dispatched by the sponsor and inspections by drug regulatory authorities to ensure the quality of the clinical trial.

I agree that the results of the study will be used for drug registration.

I will provide a resume before the start of the study, submit it to the ethics committee, and possibly submit it to the drug administration department.

Researcher (Signature):                       Date:

**Summary of changes to the protocol**

Changes from the original protocol (Version 1.0, October 15, 2013) to the final protocol (Version 3.0, September 1, 2020) are listed below:

**Page 1:**

*Previous text*: Adjuvant icotinib of 12 months or 6 months versus observation following adjuvant chemotherapy for resected EGFR-mutated stage II–IIIA non-small-cell lung cancer (ICTAN): a randomized phase III trial.

*Revised text*: Adjuvant icotinib of 12 months or 6 months versus observation following adjuvant chemotherapy for resected EGFR-mutated stage II–IIIA non-small-cell lung cancer (ICTAN, GASTO1002): a randomized phase III trial.

*Previous text*: Protocol Number: WSY003.

*Revised text*: Protocol Number: GASTO1002.

**Page 2. Title.**

*Previous text*: ICTAN.

*Revised text*: ICTAN, GASTO1002.

**Page 2. Endpoints.**

*Previous text*: Primary Endpoint: disease-free survival (DFS).

*Revised text*: Primary Endpoint: disease-free survival (DFS) according to investigator assessment.

*Previous text*: Secondary Endpoints: overall survival (OS), safety and tolerability, and quality of life (QoL).

*Revised text*: Secondary Endpoints: overall survival (OS), brain-metastasis-free survival (BMFS), safety and tolerability, and quality of life (QoL).

**Page 2. Number of cases.**

*Previous text*: 477.

*Revised text*: 318.

**Page 2. Inclusion Criteria.**

*Previous text*: Received 4 cycles of adjuvant chemotherapy postoperatively.

*Revised text*: Received 2-4 cycles of adjuvant chemotherapy postoperatively.

*Previous text*: Have not received any other chemotherapy, radiotherapy, or biotherapy except for the 4 cycles of adjuvant chemotherapy.

*Revised text*: Have not received any other chemotherapy, radiotherapy, or biotherapy except for the 2-4 cycles of adjuvant chemotherapy.

**Page 3. Inclusion Criteria.**

*Previous text*: Treatment initiation within 4 weeks after the completion of 4 cycles of adjuvant

chemotherapy.

*Revised text*: Treatment initiation within 4 weeks after the completion of 2-4 cycles of adjuvant

chemotherapy.

“Able to obtain sufficient tumor histological specimens (non-cytological) for biomarker

analysis” was deleted.

**Page 4. Study duration.**

*Previous text*: Based on an estimated enrollment of about 10 patients per month, a total of 477 patients are planned to be enrolled, which will take approximately 48 months. The minimum survival follow-up period is 5 years, making the total study duration approximately 108 months.

*Revised text*: Based on an estimated enrollment of about 5 patients per month, a total of 318 patients are planned to be enrolled, which will take approximately 64 months. The minimum survival follow-up period is 5 years, making the total study duration approximately 124 months.

**Page 5. Study groups.**

*Previous text*: After R0 resection and 4 cycles of adjuvant chemotherapy.

*Revised text*: After R0 resection and 2-4 cycles of adjuvant chemotherapy.

**Page 5. Treatment period & Follow-up period.**

*Previous text*: The planned sample size is 159 in each group.

*Revised text*: The planned sample size is 106 in each group.

**Page 6. Sample size.**

*Previous text*: A sample size of 477 (requiring 363 events) is based on the following considerations: From previous studies, it is assumed that the median DFS in for the chemotherapy-only group is 27 months, with a hazard ratio (HR) of 0.7, a power of test (1-β) of 80%, a significance level (α) of 5% for a two-sided test, and an overall dropout rate of 5%.

*Revised text*: A sample size of 318 (requiring 198 events) is based on the following considerations: From previous studies, it is assumed that the median DFS is 30 months for patients with EGFR-mutated stage II-IIIA NSCLC following adjuvant chemotherapy, with a hazard ratio (HR) of 0.6, a power of test (1-β) of 85%, a significance level (α) of 5% for a two-sided test, and an overall dropout rate of 5%.

**Page 6. Study overview.**

*Previous text*: who had undergone R0 resection and adjuvant chemotherapy for 4 cycles.

*Revised text*: who had undergone R0 resection and adjuvant chemotherapy for 2-4 cycles.

*Previous text*: N=159.

*Revised text*: N=106.

*Previous text*: Primary endpoint:

DFS

Secondary endpoints：

OS

Safety

QoL.

*Revised text*: Primary endpoint:

DFS according to investigator assessment

Secondary endpoints：

OS

BMFS

Safety

QoL.

**Page 9. List of investigators.**

"Table 2. List of investigators" was added.

**Page 10. Subject selection.**

“3.2.3 Definition of R0 resection” was added.

**Page 15. 2.1. Primary objective.**

*Previous text*: Disease-free survival (DFS).

*Revised text*: Disease-free survival (DFS) according to investigator assessment.

**Page 15. 2.2. Secondary objectives.**

*Previous text*: Overall survival (OS), safety and tolerability, and quality of life (QoL).

*Revised text*: Overall survival (OS), brain-metastasis-free survival (BMFS), safety and tolerability, and quality of life (QoL).

**Page 15. 3.1. Study design and plan.**

*Previous text*: A sample size of 477 (requiring 363 events) is based on the following considerations: From previous studies, it is assumed that the median DFS in the chemotherapy-only group is 27 months, with a hazard ratio (HR) of 0.7, a power of test (1-β) of 80%, a significance level (α) of 5% for a two-sided test, and an overall dropout rate of 5%. These 477 patients are planned to be enrolled in the study.

*Revised text*: A sample size of 318 (requiring 198 events) is based on the following considerations: From previous studies, it is assumed that the median DFS is 30 months for patients with EGFR-mutated stage II-IIIA NSCLC following adjuvant chemotherapy, with a hazard ratio (HR) of 0.76, a power of test (1-β) of 85%, a significance level (α) of 5% for a two-sided test, and an overall dropout rate of 5%. These 318 patients are planned to be enrolled in the study.

**Page 16. 3.2.1. Inclusion criteria.**

*Previous text*: Received 4 cycles of adjuvant chemotherapy postoperatively.

*Revised text*: Received 2-4 cycles of adjuvant chemotherapy postoperatively.

“Able to obtain sufficient tumor histological specimens (non-cytological) for biomarker

analysis” was deleted.

**Page 18.**

“3.2.3. Definition of R0 resection:

Hilar and mediastinal lymph nodes (N1 and N2 lymph nodes) must be excised, marked, and sent for pathology in addition to complete removal of the primary lung lesions. At least 3 mediastinal drainage areas (N2 groups) must be sampled and dissected by making the best use of en bloc resection of lymph nodes. Preferably, for the right chest, dissection should include 2R, 3a, 3p, 4R, and 7-9 groups of lymph nodes and surrounding soft tissues; and for the left chest, dissection should include 4L and 5-9 groups of lymph nodes and surrounding soft tissues” was added.

**Page 18. 3.3. Study duration.**

*Previous text*: It is planned to enroll a total of 477 patients, and based on an estimated enrollment of about 10 patients per month, the enrollment process is expected to take approximately 48 months; the minimum survival follow-up period is 5 years. The total study period is expected to be about 108 months.

*Revised text*: It is planned to enroll a total of 318 patients, and based on an estimated enrollment of about 5 patients per month, the enrollment process is expected to take approximately 64 months; the minimum survival follow-up period is 5 years. The total study period is expected to be about 124 months.

**Page 21. 3.6. Study endpoint.**

*Previous text*: The primary efficacy endpoint of the study is DFS.

*Revised text*: The primary efficacy endpoint of the study is DFS according to investigator assessment.

*Previous text*: Secondary endpoints include: OS, safety and tolerability, and QoL.

*Revised text*: Secondary endpoints include: OS, BMFS, safety and tolerability, and QoL.

**Page 30. 8.1. Primary and secondary variables.**

*Previous text*: The primary efficacy endpoint of the study is DFS.

*Revised text*: The primary efficacy endpoint of the study is DFS according to investigator assessment.

*Previous text*: Secondary endpoints include: OS, safety and tolerability, and QoL.

*Revised text*: Secondary endpoints include: OS, safety and tolerability, and QoL.

**Page 31. 8.2.1. Baseline and demographic characteristics.**

“More details will be stated in the separate Statistical Analysis Plan” was added.

**Page 31. 8.2.2. Primary efficacy.**

*Previous text*: The primary efficacy variable of this study is the DFS.

*Revised text*: The primary efficacy variable of this study is the DFS according to investigator assessment.

**Page 31. 8.2.3. Secondary efficacy.**

“BMFS” was added.

**Page 32. 8.4. Interim analysis.**

*Previous text*: Interim analysis will be conducted when the DFS events reach 50% (182/363) for final analysis.

*Revised text*: Interim analysis will be conducted when the DFS events reach 50% (99/198) for final analysis. More details about the statistical methods will be stated in the separate Statistical Analysis Plan.

**Page 32. 8.5. Safety analysis.**

*Previous text*:

- Adverse Events;
- Serious Adverse Events;
- Laboratory Parameters;
- Vital signs, including ECOG performance status.

*Revised text*:

- Adverse Events;
- Serious Adverse Events;
- All adverse events ≥ grade 3;
- All adverse events leading to treatment discontinuation;
- All adverse events leading to dose reduction;
- Fatal adverse events.
