# Supplementary material for: Adjuvant icotinib for resected EGFR-mutated stage II–IIIA non-small-cell lung cancer (ICTAN, GASTO1002): a randomized comparison study
Source: Signal Transduct Target Ther. 2025 Aug 28;10:273. doi: 10.1038/s41392-025-02358-w (PMC12391381; doi:10.1038/s41392-025-02358-w)
Supplement: Supplementary file 3 — Statistical Analysis Plan [file 41392_2025_2358_MOESM3_ESM.docx]

**STATISTICAL ANALYSIS PLAN**

**Adjuvant icotinib of 12 months or 6 months versus observation following adjuvant chemotherapy for resected EGFR-mutant stage II–IIIA non-small-cell lung cancer (ICTAN, GASTO1002): a randomized phase 3 trial.**

**Principal investigator: Si-Yu Wang**

**Sponsor: Betta Pharmaceuticals Co., Ltd.**

**Registration No.: NCT01996098**

**Version: 3.0, September 1, 2020.**

**Confidentiality Statement**

This confidential document is the property of the sponsor. No unpublished information contained in this document may be disclosed without prior written approval of the sponsor.

**Table of contents**

[Title page 1](#_Toc170380163)

[Table of contents 2](#_Toc170380164)

[List of abbreviations 4](#_Toc170380165)

[1. Introduction 5](#_Toc170380166)

[2. Study objectives 5](#_Toc170380168)

[2.1. Primary objective 5](#_Toc170380169)

[2.2. Secondary objectives 5](#_Toc170380170)

[2.3. Exploratory objectives 5](#_Toc170380171)

[3. Study design and randomization 6](#_Toc170380172)

[3.1. Study design 6](#_Toc170380173)

[3.2. Randomization 7](#_Toc170380174)

[3.3. Blinding 7](#_Toc170380174)

[4. Sample size 7](#_Toc170380196)

[5. Analysis sets 8](#_Toc170380201)

[5.1. Intention-to-treat set 8](#_Toc170380202)

[5.2. Per-protocol set 8](#_Toc170380202)

[5.3. Safety analysis set 8](#_Toc170380203)

[5.4. Subgroup analysis set 8](#_Toc170380204)

[6. Drug exposure 8](#_Toc170380213)

[7. Study endpoints 9](#_Toc170380214)

[7.1. Efficacy endpoints 9](#_Toc170380216)

[*7.1.1. Primary efficacy endpoint 9*](#_Toc170380217)

[*7.1.2. Secondary efficacy endpoints 9*](#_Toc170380218)

[7.2. Safety endpoints 10](#_Toc170380219)

[*7.2.1. Adverse events 10*](#_Toc170380220)

[*7.2.2. Serious adverse events 10*](#_Toc170380221)

[7.3. Quality of life (QoL)....................................................................... 11](#_Toc170380224)

[8. Category and definition of recurrence 11](#_Toc170380227)

[8.1. Category of recurrence 11](#_Toc170380228)

[8.2. Definition of local and distant recurrence 11](#_Toc170380229)

[9. Baseline patient characteristics 11](#_Toc170380241)

[10. Interim analysis 12](#_Toc170380241)

[11. Handling missing data 12](#_Toc170380241)

[12. Statistical considerations 12](#_Toc170380242)

[13. References 13](#_Toc170380242)

[14. Appendix 14](#_Toc170380242)

[15. Signature page 15](#_Toc170380242)

[16. Summary of changes to the Statistical Analysis Plan 16](#_Toc170380242)

List of abbreviations

| **Terms** | **Definition** |
| --- | --- |
| AE | Adverse event |
| AJCC | American Joint Committee on Cancer |
| ALT | Alanine aminotransferase |
| AST | Aspartate aminotransferase |
| BMFS | Brain-metastasis-free survival |
| CI | Confidence interval |
| CT | Computer tomography |
| CTCAE | Common Terminology Criteria for Adverse Events |
| DFS | Disease-free survival |
| ECG | Electrocardiogram |
| ECOG PS | Eastern Cooperative Oncology Group performance status |
| EGFR | Epidermal growth factor receptor |
| FACT-L | Functional Assessment of Cancer Therapy—Lung Cancer |
| GASTO | Guangdong Association of Study of Thoracic Oncology |
| HR | Hazard ratio |
| IDMC | Independent Data Monitoring Committee |
| QoL | Quality of life |
| ITT | Intention-to-treat |
| KM | Kaplan-Meier |
| LCSS | Lung Cancer Symptom Scale |
| MRI | Magnetic resonance imaging |
| NCI | National Cancer Institute |
| NSCLC | Non-small-cell lung cancer |
| OS | Overall survival |
| PD-1 | Programmed death receptor 1 |
| PD-L1 | Programmed death-ligand 1 |
| PFS | Progression-free survival |
| RECIST | Response Evaluation Criteria in Solid Tumors |
| SAE | Serious adverse event |
| TID | ter in die; three times a day |
| TKI | Tyrosine kinase inhibitor |

**1. Introduction**

This Statistical Analysis Plan describes the statistical analysis methods to be used in the presentation and analysis of efficacy and safety data for the ICTAN trial (NCT01996098). ICTAN is a randomized open-label phase 3 trial assessing the efficacy and safety of adjuvant icotinib for 12 months or 6 months versus observation following adjuvant chemotherapy for resected EGFR-mutant stage II-IIIA NSCLC.

This study’s statistical analysis plan is prepared based on trial protocol version 3.0 (September 1, 2020).

**2. Study objectives**

**2.1. Primary objective**

To evaluate the efficacy of adjuvant 12-month or 6-month icotinib compared with observation, in terms of disease-free survival (DFS), according to investigator assessment, for patients with completely resected EGFR-mutant stage II-IIIA non-small-cell lung cancer (NSCLC) after adjuvant chemotherapy.

**2.2. Secondary objectives**

To evaluate the efficacy of adjuvant 12-month or 6-month icotinib compared with observation, in terms of overall survival (OS), for patients with completely resected EGFR-mutant stage II-IIIA NSCLC after adjuvant chemotherapy.

To evaluate the efficacy of adjuvant 12-month or 6-month icotinib compared with observation, in terms of brain-metastasis-free survival (BMFS).

To evaluate the safety and tolerability of adjuvant 12-month or 6-month icotinib for patients with completely resected EGFR-mutant stage II-IIIA NSCLC after adjuvant chemotherapy, as compared with observation.

To evaluate the quality of life (QoL) of adjuvant 12-month or 6-month icotinib compared with observation.

To evaluate the efficacy of adjuvant 12-month icotinib compared with 6-month icotinib, in terms of DFS, OS and BMFS, for patients with completely resected EGFR-mutant stage II-IIIA NSCLC after adjuvant chemotherapy.

To evaluate the safety and tolerability of adjuvant 12-month icotinib compared with 6-month icotinib for patients with completely resected EGFR-mutant stage II-IIIA NSCLC after adjuvant chemotherapy.

To evaluate the QoL of adjuvant 12-month icotinib compared with 6-month icotinib for patients with completely resected EGFR-mutant stage II-IIIA NSCLC after adjuvant chemotherapy.

**2.3. Exploratory objectives**

Biomarker exploration.

**3. Study design and randomization**

**3.1. Study design**

ICTAN is a randomized open-label phase 3 trial assessing the efficacy and safety of adjuvant icotinib for 12 months or 6 months versus observation following adjuvant chemotherapy for resected EGFR-mutant stage II-IIIA NSCLC.

Eligible patients after screening

12-month icotinib group

6-month icotinib group

Observation group

Follow-up

Primary endpoint：

DFS according to investigator assessment

Secondary endpoints：

OS

BMFS

Safety

QoL

Patients with pathological stage II-IIIA EGFR-mutant NSCLC after adjuvant chemotherapy

Eligible patients will be randomly assigned in a 1:1:1 ratio to receive icotinib (125mg, three times daily) for 12 months, icotinib for 6 months, or to undergo observation. Therapy will be continued until disease progression or intolerable toxicity occurs. The first dose of study treatment should be taken no later than 7 days after randomization.

**3.2. Randomization**

The randomization scheme was produced by the central staff of the ICTAN trial via a computer-generated sequence with a minimization method. Randomization was stratified according to sex (male vs female) and stage (II vs IIIA). Eligible patients were randomized at a ratio of 1:1:1 to receive icotinib for 12 months, icotinib for 6 months, or to undergo observation. Randomization will be performed just before the initiation of study administration.

**3.3. Blinding**

This is an open-label study. All patients, investigators, and staff involved in the study were not blinded to the treatment assignment.

**4. Sample size**

Approximately 318 patients will be enrolled in this study. The sample size is based on the following considerations:

Based on the LACE analysis and the BR19 trial ^1,2^, we assumed that the median DFS is 30 months for patients with EGFR-mutant stage IIA-IIIA NSCLC following adjuvant chemotherapy;

We aimed to achieve 85% power at a two-sided α of 0.05 and an overall dropout rate of 5%;

A total of 318 patients are needed to detect a 40% improvement in DFS with icotinib compared with chemotherapy for each comparison: 12 months of icotinib versus observation and 6 months of icotinib versus observation;

This improvement corresponds to a hazard ratio (HR) of 0.6;

A total of 198 DFS events are required for the final analysis.

**5. Analysis sets**

**5.1. Intention-to-treat set.**

The Intention-To-Treat (ITT) set will consist of all randomized subjects. The ITT population will be used for the efficacy analysis of DFS, OS and BMFS.

**5.2. Per-protocol set.**

The per-protocol (PP) set will consist of patients who complete planned treatments, including patients who complete 12 or 6 months of icotinib, and patients in the observation group. The PP population will be used for the efficacy analysis of DFS and OS.

**5.3. Safety analysis set.**

The safety analysis set will include all subjects who receive at least 1 dose of the study drug and those in the observation group. All subjects in the safety analysis set will have at least a safety record after randomization.

**5.4. Subgroup analysis set.**

Subgroup analyses will be conducted comparing DFS based on the following pre-planned factors:

**•** Sex (male vs. female);

**•** Pathological stage (II vs IIIA);

**•** EGFR mutation (exon 19 deletion vs. exon 21 L858R).

Other stratification factors, including age (≥65 vs <65), smoking history (ever vs never), and N stage (0 and 1 vs 2), may also be assessed. Subgroup analyses for OS will be performed according to the above stratification factors.

6. Drug exposure

Days of exposure = date of the last dose (dose > 0 mg) – date of the first dose + 1.

Actual days of exposure = (end date of study drug 1 - start date of study drug 1 + 1) + (end date of study drug 2 - start date of study drug 2 + 1) + … + (end date of study drug n - start date of study drug n + 1); dose > 0 mg.

Proportion of patients with exposure time achieving ＜1 month, 1-2 months, 2-3 months, 3-4 months, 4-5 months, 5-6 months, 6-7 months, 7-8 months, 8-9 months, 9-10 months, 10-11 months, 11-12 months, 12-13 months, 13-18 months, and ≥ 18 months during icotinib treatment will be recorded.

Dose modification, including dose reduction and drug interruption, and reasons for dose modification will be recorded.

**7. Study endpoints**

**7.1. Efficacy endpoints**

**7.1.1. Primary efficacy endpoint**

The primary endpoint is DFS, which is defined as the time from randomization to disease recurrence or death, whichever occurs first. DFS time is calculated as:

Follow-up time for DFS (days) = date of recurrence (patients who had recurrence) or date of the last follow-up – date of randomization + 1.

Patients who are disease-free and alive at the time of analysis will be censored at the date of their last follow-up. Three-year and 5-year DFS are defined as the proportion of patients who are alive and disease-free at 3 years and 5 years after randomization.

**7.1.2. Secondary efficacy endpoints**

The secondary efficacy endpoints included OS and BMFS. OS is defined as the time from random assignment to death from any cause. Patients who are alive at the time of analysis will be censored at the date of their last follow-up. Three-year and 5-year OS are defined as the proportion of patients who are alive at 3 years and 5 years after randomization. OS time is calculated as:

Follow-up time for OS (days) = date of death (patients who died) or date of the last follow-up – date of randomization + 1.

BMFS is defined as the time from randomization to brain metastasis or death, whichever comes first. Three-year and 5-year BMFS are defined as the proportion of patients who are alive and brain-metastasis-free at 3 years and 5 years after randomization. BMFS time is calculated as:

Follow-up time for BMFS (days) = date of brain metastases (patients who had brain metastases) or date of the last follow-up – date of randomization + 1.

**7.2. Safety endpoints**

**7.2.1. Adverse events**

All patients who receive at least 1 dose of study treatment in the 12-month or 6-month icotinib group and all those in the observation group will be evaluated for safety and tolerability. Safety and tolerability will be evaluated from informed consent to every visit. The investigator is responsible for evaluating the relationship between all adverse events and the study drug. Adverse events should be classified and graded according to the NCI Common Terminology Criteria for Adverse Events (CTCAE) Version 4.0.

The following summaries will be calculated for adverse events:

**•** All adverse events;

**•** All adverse events ≥ grade 3;

**•** All adverse events leading to treatment discontinuation;

**•** All adverse events leading to dose reduction;

• Fatal adverse events.

**7.2.2. Serious adverse events**

Serious adverse events are all unfavorable adverse events occurring during the study that meet at least one of the following criteria:  **•** Death; **•** Life-threatening conditions; **•** Resulting in patient’s hospitalization or prolonged hospitalization of inpatients;
• Leading to persistent or significant incapacity or disability;
• Important Medical Events that may require medical intervention to prevent one of the conditions mentioned above.

**7.3 Quality of life (QoL)**

QoL will be assessed with the Functional Assessment of Cancer Therapy – Lung (FACT-L) questionnaire and Lung Cancer Symptom Scale (LCSS).

**8. Category and definition of recurrence**

**8.1. Category of recurrence.**

Recurrence will be categorized as local only, distant only or both local and distant.

**8.2. Definition of local and distant recurrence.**

Local recurrence is defined as the relapse in the lung, regional lymph nodes and pleura. Distant recurrence is defined as the spread of disease beyond the area of the lung, regional lymph nodes and pleura, including the brain, liver and bone.

**9. Baseline patient characteristics**

Patient demographics will be listed and summarized at baseline. The categorical variables will include the following:

• Age (years; median, minimum, maximum)

Age is calculated as:

Age (years) = (date of informed consent - date of birth +1)/365.

• Sex (male, female)

• ECOG PS (0, 1)

• Smoking status (current, former, never)

• Histology (adenocarcinoma, squamous cell carcinoma, other)

• Pathological stage according to 7th AJCC staging ^3^ (IIA, IIB, IIIA)

• Tumor stage (T1, T2, T3, T4)

• N stage (N0, N1, N2)

• Surgery type (lobectomy, pneumonectomy, other)

• Side (left, right)

• EGFR mutational status (exon 19 deletion, exon 21 L858R)

• Adjuvant therapies

Cycles and regimens of adjuvant chemotherapy for NSCLC will be summarized. Medical history and concomitant medications will be recorded.

**10. Interim Analysis**

Interim analysis will be conducted when the DFS events reach 50% (99/198) for final analysis. Futility analysis will be performed in the interim analysis. The timing and frequency of the interim analyses may be changed if the Independent Data Monitoring Committee (IDMC) considers it necessary. The IDMC will make recommendations to continue, amend, or stop the study based on serious adverse events, adverse events, and other safety data. If the interim analysis meets the criteria of early termination of the trial, the sponsor and the principal investigator may accept IDMC's recommendation to terminate the study. Otherwise, the study will continue with or without sample size adjustment.

**11. Handling missing data**

With the exception of dates, imputation for missing data will not be performed principally. For efficacy analyses, if a subject’s month and year of an event are provided but the day is missing, the day will be set to the first day of the month.

**12. Statistical Considerations**

Descriptive statistics will be defined for continuous variables as number (n), median, mean, minimum, maximum, and 25th and 75th percentiles. Categorical variables will be summarized through frequencies and percentages.

The Kaplan-Meier method is used to estimate time-to-event endpoints, with differences compared by log-rank tests with two-sided p values ^4^. The Cox proportional hazards model is used to estimate hazard ratios (HRs), 95% confidence intervals (CIs), and Wald P values ^5^.

Unless otherwise specified, all statistical analyses will be performed through two-sided tests with a significance level of 0.05. All statistical analyses will be performed through SPSS and R.

Month=365/12 days.

Year=365 days.

**13. References**

1 Pignon, J. P. *et al.* Lung adjuvant cisplatin evaluation: a pooled analysis by the LACE Collaborative Group. *J Clin Oncol* **26**, 3552-3559, doi:10.1200/JCO.2007.13.9030 (2008).

2 Goss, G. D. *et al.* Gefitinib versus placebo in completely resected non-small-cell lung cancer: results of the NCIC CTG BR19 study. *J Clin Oncol* **31**, 3320-3326, doi:10.1200/JCO.2013.51.1816 (2013).

3 Goldstraw, P. *et al.* The IASLC Lung Cancer Staging Project: proposals for the revision of the TNM stage groupings in the forthcoming (seventh) edition of the TNM Classification of malignant tumours. *J Thorac Oncol* **2**, 706-714, doi:10.1097/JTO.0b013e31812f3c1a (2007).

4 Kaplan, E. L. & Meier, P. Nonparametric estimation of incomplete observations. *J Amer Stat Assoc.* **53**, 457-481 (1958).

5 Cox, D. Regression models and life-tables. *J Royal Stat Soc Ser B.* **34**, 187-220 (1972).

**14. Appendix. ECOG PS criteria.**

| Score | Criteria | Description |
| --- | --- | --- |
| 0 | Asymptomatic | Fully active, able to carry on all predisease activities without restriction. |
| 1 | Symptomatic but completely ambulatory | Restricted in physically strenuous activity but ambulatory and able to carry out work of a light or sedentary nature. For example, light housework and office work. |
| 2 | Symptomatic, <50% in bed during the day | Ambulatory and capable of all self care but unable to carry out any work activities. Up and about more than 50% of waking hours. |
| 3 | Symptomatic, >50% in bed, but not bedbound | Capable of only limited self-care, confined to bed or chair 50% or more of waking hours. |
| 4 | Bedbound | Completely disabled. Cannot carry on any self-care. Totally confined to bed or chair. |
| 5 | Death |  |

**15. Signature page**

| Document title: Statistical Analysis Plan Version 3.0 | |
| --- | --- |
| Trial name: Adjuvant icotinib of 12 months or 6 months versus observation following adjuvant chemotherapy for resected EGFR-mutant stage II–IIIA non-small-cell lung cancer (ICTAN, GASTO1002): a randomized phase 3 trial. | |
| Version: 3.0 | |
| Document date: September 1, 2020 | |
| Investigator: | |
| Signature: | Date: |

**16. Summary of changes to the Statistical Analysis Plan**

Changes from the original Statistical Analysis Plan (Version 1.0, October 15, 2013) to the final Statistical Analysis Plan (Version 3.0, September 1, 2020) are listed below:

**Page 1:**

*Previous text:* Study: ICTAN.

*Revised text:* Study: ICTAN, GASTO1002.

*Previous text:* Adjuvant icotinib of 12 months or 6 months versus observation following adjuvant chemotherapy for resected EGFR-mutated stage II–IIIA non-small-cell lung cancer (ICTAN): a randomized phase III trial.

*Revised text:* Adjuvant icotinib of 12 months or 6 months versus observation following adjuvant chemotherapy for resected EGFR-mutated stage II–IIIA non-small-cell lung cancer (ICTAN, GASTO1002): a randomized phase III trial.

**Page 5. 2.1. Primary objective.**

*Previous text:* To evaluate the efficacy of adjuvant 12-month or 6-month icotinib compared with observation, in terms of disease-free survival (DFS), for patients with completely resected EGFR-mutant stage II-IIIA non-small-cell lung cancer (NSCLC) after adjuvant chemotherapy.

*Revised text:* To evaluate the efficacy of adjuvant 12-month or 6-month icotinib compared with observation, in terms of disease-free survival (DFS), according to investigator assessment, for patients with completely resected EGFR-mutant stage II-IIIA non-small-cell lung cancer (NSCLC) after adjuvant chemotherapy.

**Page 5. 2.2. Secondary objectives.**

“To evaluate the efficacy of adjuvant 12-month or 6-month icotinib compared with observation, in terms of brain-metastasis-free survival (BMFS)” was added.

*Previous text:* To evaluate the efficacy of adjuvant 12-month icotinib compared with 6-month icotinib, in terms of DFS and OS, for patients with completely resected EGFR-mutant stage II-IIIA NSCLC after adjuvant chemotherapy.

*Revised text:* To evaluate the efficacy of adjuvant 12-month icotinib compared with 6-month icotinib, in terms of DFS, OS and BMFS, for patients with completely resected EGFR-mutant stage II-IIIA NSCLC after adjuvant chemotherapy.

**Page 6. 3.1. Study design.**

*Previous text:* Primary endpoint:

DFS

Secondary endpoints：

OS

Safety

QoL.

*Revised text:* Primary endpoint:

DFS according to investigator assessment

Secondary endpoints：

OS

BMFS

Safety

QoL.

**Page 7. 4. Sample size.**

*Previous text:* Approximately 477 patients will be enrolled in this study.

*Revised text:* Approximately 318 patients will be enrolled in this study.

*Previous text:* We assumed that the median DFS is 27 months for patients with EGFR-mutant stage IIA-IIIA NSCLC following adjuvant chemotherapy.

*Revised text:* Based on the LACE analysis and the BR19 trial, we assumed that the median DFS is 30 months for patients with EGFR-mutant stage IIA-IIIA NSCLC following adjuvant chemotherapy.

*Previous text:* We aimed to achieve 80% power at a two-sided α of 0.05 and an overall dropout rate of 5%. A total of 477 patients are needed to detect a 30% improvement in DFS with icotinib compared with chemotherapy for each comparison: 12 months of icotinib versus observation and 6 months of icotinib versus observation;

This improvement corresponds to a hazard ratio (HR) of 0.7;

A total of 363 DFS events are required for the final analysis.

*Revised text:* We aimed to achieve 85% power at a two-sided α of 0.05 and an overall dropout rate of 5%. A total of 318 patients are needed to detect a 40% improvement in DFS with icotinib compared with chemotherapy for each comparison: 12 months of icotinib versus observation and 6 months of icotinib versus observation;

This improvement corresponds to a hazard ratio (HR) of 0.6;

A total of 198 DFS events are required for the final analysis.

**Page 9. 7.1.2. Secondary efficacy endpoints.**

*Previous text:* The secondary efficacy endpoints was OS.

*Revised text:* The secondary efficacy endpoints included OS and BMFS.

**Page 10. 7.1.2. Secondary efficacy endpoints.**

“BMFS is defined as the time from randomization to brain metastasis or death, whichever comes first. Three-year and 5-year BMFS are defined as the proportion of patients who are alive and brain-metastasis-free at 3 years and 5 years after randomization. BMFS time is calculated as:

Follow-up time for BMFS (days) = date of brain metastases (patients who had brain metastases) or date of the last follow-up – date of randomization + 1” was added.

**Page 12. 10. Interim analysis.**

*Previous text:* Interim analysis will be conducted when the DFS events reach 50% (182/363) for final analysis.

*Revised text:* Interim analysis will be conducted when the DFS events reach 50% (99/198) for final analysis.

**Page 15. 15. Signature page**

*Previous text:* Adjuvant icotinib of 12 months or 6 months versus observation following adjuvant chemotherapy for resected EGFR-mutant stage II–IIIA non-small-cell lung cancer (ICTAN): a randomized phase 3 trial.

*Revised text:* Adjuvant icotinib of 12 months or 6 months versus observation following adjuvant chemotherapy for resected EGFR-mutant stage II–IIIA non-small-cell lung cancer (ICTAN, GASTO1002): a randomized phase 3 trial.
